# Microphysiological systems for solid tumor immunotherapy: opportunities and challenges

**DOI:** 10.1038/s41378-023-00616-x

**Published:** 2023-12-15

**Authors:** Sara Abizanda-Campo, María Virumbrales-Muñoz, Mouhita Humayun, Ines Marmol, David J. Beebe, Ignacio Ochoa, Sara Oliván, Jose M. Ayuso

**Affiliations:** 1 Department of Dermatology, University of Wisconsin-Madison, Madison, WI USA; 2 University of Wisconsin Carbone Cancer Center, Madison, WI USA; 3 Department of Biomedical Engineering, University of Wisconsin, Madison, WI USA; 4 Tissue Microenvironment Lab (TME lab), Aragón Institute of Engineering Research (I3A), University of Zaragoza, Zaragoza, Spain; 5 Instituto de Investigación Sanitaria Aragón (IISA), Zaragoza, Spain; 6 Centro Investigación Biomédica en Red. Bioingeniería, Biomateriales y Nanomedicina (CIBER-BBN), Zaragoza, Spain; 7 Department of Obstetrics and Gynecology, University of Wisconsin-Madison, Madison, WI USA; 8 Department of Biological Engineering, Massachusetts Institute of Technology Cambridge, Cambridge, MA USA; 9 Department of Pathology & Laboratory Medicine, University of Wisconsin, Madison, WI USA

**Keywords:** Nanofluidics, Engineering

## Abstract

Immunotherapy remains more effective for hematologic tumors than for solid tumors. One of the main challenges to immunotherapy of solid tumors is the immunosuppressive microenvironment these tumors generate, which limits the cytotoxic capabilities of immune effector cells (e.g., cytotoxic T and natural killer cells). This microenvironment is characterized by hypoxia, nutrient starvation, accumulated waste products, and acidic pH. Tumor-hijacked cells, such as fibroblasts, macrophages, and T regulatory cells, also contribute to this inhospitable microenvironment for immune cells by secreting immunosuppressive cytokines that suppress the antitumor immune response and lead to immune evasion. Thus, there is a strong interest in developing new drugs and cell formulations that modulate the tumor microenvironment and reduce tumor cell immune evasion. Microphysiological systems (MPSs) are versatile tools that may accelerate the development and evaluation of these therapies, although specific examples showcasing the potential of MPSs remain rare. Advances in microtechnologies have led to the development of sophisticated microfluidic devices used to recapitulate tumor complexity. The resulting models, also known as microphysiological systems (MPSs), are versatile tools with which to decipher the molecular mechanisms driving immune cell antitumor cytotoxicity, immune cell exhaustion, and immune cell exclusion and to evaluate new targeted immunotherapies. Here, we review existing microphysiological platforms to study immuno-oncological applications and discuss challenges and opportunities in the field.

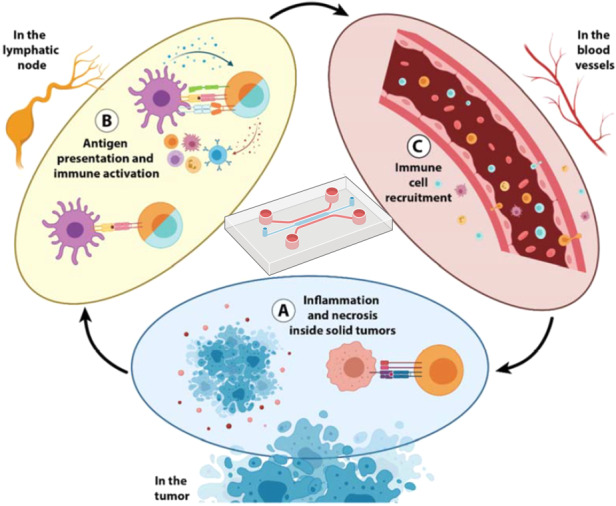

## Introduction: the current landscape of cancer immunotherapy

**Fig. 1:**
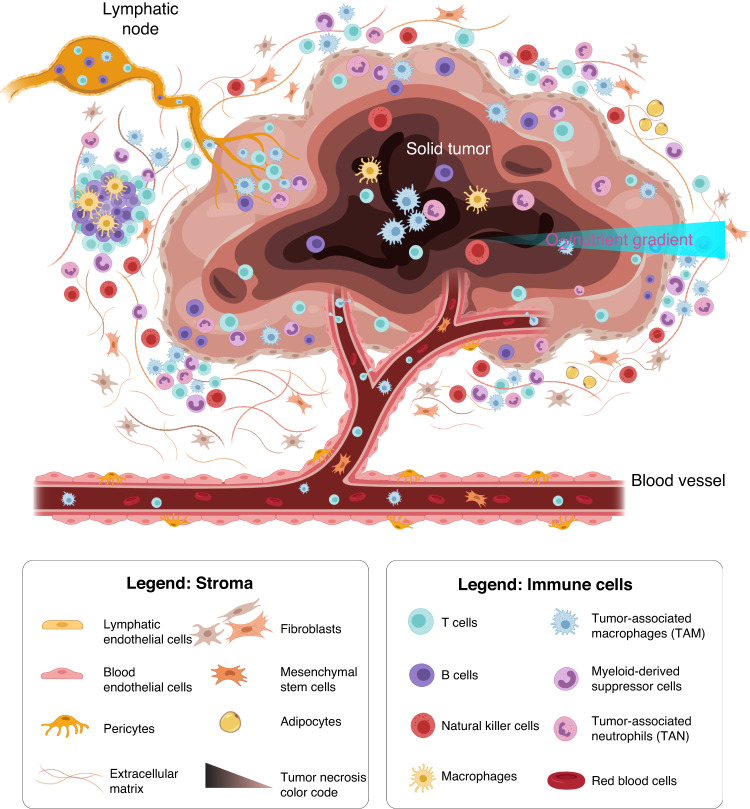
Immune microenvironment in a solid tumor. Solid tumors are complex structures where cellular (e.g., cancer, stromal, immune), molecular (e.g., cytokines), and biochemical (e.g., oxygen and nutrients) factors interact and exert a profound impact on the antitumor immune response

In recent years, breakthroughs in immunology have pushed immunotherapies into the role of first-line treatment for several tumor types^1–3^. The discovery of immune checkpoints has led to the development of a new type of inhibitor (e.g., antibodies targeting the PD-1/PD-L1 axis and CTLA-4) that prevents the tumor from blocking the immune response^4–6^. As a consequence, immune checkpoint inhibitors (ICIs) are among first-line treatments used against multiple tumors^7–9^, the discovery of ICIs warranted a Nobel prize in 2018^10^. New targets, including LAG-3, TIM-3, B7-H3, and B7-H4, are being increasingly explored for their potential in immune checkpoint blockade^8,11–13^. In parallel, advances in genetic engineering and cellular immunology have led to the generation of chimeric antigen receptor (CAR) T or natural killer (NK) cells. CAR T or NK cells are genetically engineered to stably express a tumor antigen receptor, which after immune activation, triggers a cytotoxic response and leads to tumor cell lysis^14–17^. Clinical trials evaluating CAR T cells for use in treating hematological cancers showed promising results (e.g., CAR T cells led to a 80–90% complete response in acute lymphoblastic leukemia patients, compared with a 20–30% response induced by traditional chemotherapy)^18,19^. These results led to rapid FDA approval of CAR T-cell therapy (i.e., Kymriah®, developed by Novartis, was approved in 2017). Because of the encouraging results of CAR T-cell therapy, the number of clinical trials evaluating immunotherapy has skyrocketed (>1000 clinical trials were recruiting in 2022), fueling an effort to extrapolate the successes of immunotherapy against hematologic cancers to solid tumors^20–22^. However, solid tumors present unique challenges to immunotherapy^23–25^ since they can disrupt the immune response by fostering an immunosuppressive environment that enhances tumor cell immune evasion and tumor growth^26,27^. Specifically, cancer cells recruit regulatory T cells (Tregs), a subset of CD4 T cells that downregulate tumor antigen expression, induce T-cell tolerance and/or apoptosis, and produce immunosuppressive cytokines that stimulate inhibitory immune checkpoint activity^28–30^. This cascade of events results in a unique and highly immunosuppressive tumor microenvironment (TME)^13,31,32^ that hinders the immune response (i.e., immune evasion). Thus, when immune cells are recruited to a tumor from nearby vessels and migrate through 3D tissue to engage with tumor cells, they enter an environment with a compromised blood supply and characterized by nutrient starvation, hypoxia, acidic pH, and waste product accumulation^33,34^. Numerous cell types found at tumor sites, including stromal cells, immune cells, fibroblasts, and endothelial cells, also condition the tumor microenvironment^35,36^ (Fig. 1). Altogether, these environmental factors severely dampen or even block the immune response and present an opportunity for improving the efficacy of immunotherapies.

To overcome the challenges posed by the immunosuppressive TME to the efficacy of immunotherapy, the mechanisms driving immune escape, including immune cell exhaustion and tumor cell evasion, have become a recent area of interest. However, traditional in vitro models fail to recapitulate the multitude of cellular, physical, and biochemical cues in the TME^37^, and these cues are key to the mechanisms underlying tumor immune escape^38,39^. The lack of in vitro models recapitulating TME cues has been a major obstacle to the translation of experimental results into the clinic^40,41^. In contrast, animal models suffer from profound interspecies differences that limit the translatability of results, not to mention ethical concerns associated with their use^42,43^. Specifically, the utility of mouse models as the quintessential model in immunology is limited by the significant cellular and molecular differences in both innate and adaptive immunity between humans and mice, which are reviewed in detail elsewhere^44,45^. For example, mouse neutrophils account for 10–25% of circulating immune cells, whereas human neutrophils represent 50–70% of the total number of immune cells. Moreover, the numerous unconserved pathways of human and mouse immunity indicate key differences in their molecular composition. Notably, granulysin is a potent cytolytic and proinflammatory cytokine that is required for the effector function (i.e., killing of tumor cells) of human cytotoxic cells (i.e., T and NK cells) but is absent in mice^44^. Sustained IFN-γ secretion leads to demyelization in the human brain, contributing to multiple tissue damage (e.g., sclerosis progression), whereas in animal models, IFN-γ seems to protect the myelin shaft and slow disease progression^46^. Finally, humans display a larger receptor repertoire, including antibody receptors (e.g., Fc-gamma-R1) and Toll-like receptors, than mice. These receptors allow immune cells to recognize their targets (e.g., tumor cells) via a variety of mechanisms, increasing their capacity to respond to tumor cells and pathogens. Thus, in vitro models capable of overcoming these limitations are needed to increase the translatability of preclinical results and thus improve tumor immunotherapy outcomes^45^.

This review lays out the basic mechanisms of immune antitumor activity (i.e., the cancer-immunity cycle), highlighting the key steps that might be made more effective with in vitro models that more closely resemble humans. We provide a more in-depth view of some of these steps and some of the opportunities for new microfluidics-based models (e.g., MPSs) to improve solid tumor immunotherapies.

## The cancer-immunity cycle

**Fig. 2:**
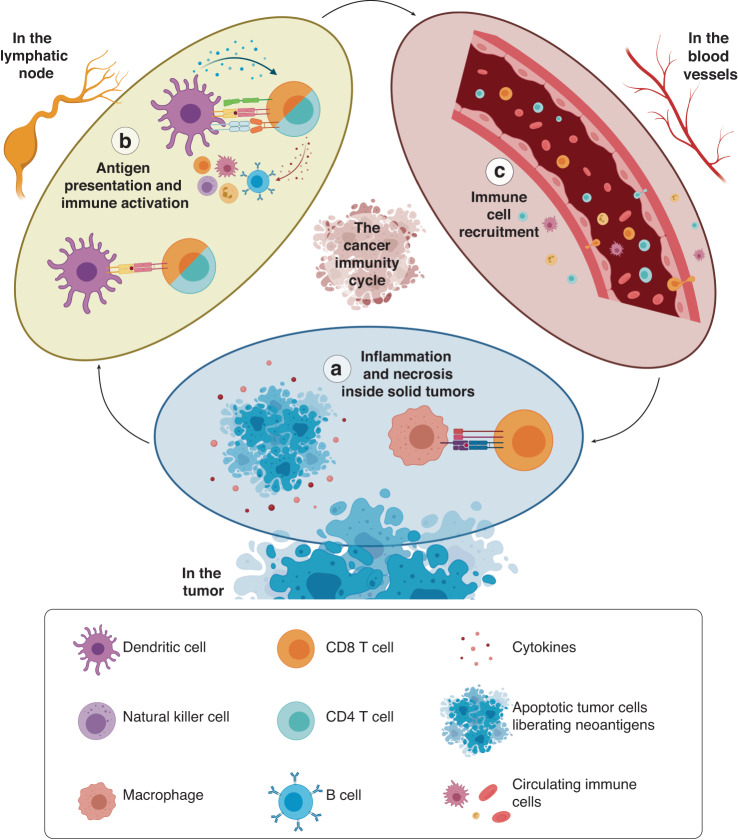
The cancer–immunity cycle. **a** Tumor mutations and aberrant cell functions generate tumor-specific neoantigens. Cancer cell death within solid malignancies releases neoantigens into the tumor milieu. Tumor neoantigens are captured and processed by antigen-presenting cells (APCs) (e.g., DCs). **b** APCs travel to lymph nodes to present tumor antigens to T cells, activating the T cells. Activated T cells enter the bloodstream and circulate through blood vessels. **c** Activated T cells recognize the inflamed endothelium, extravasate the vasculature and infiltrate tissue. Activated T cells navigate the tumor microenvironment (back to **a**) and engage with tumor cells. Activated T cells recognize the tumor neoantigens expressed on the tumor cell membrane and elicit a cytotoxic response, killing tumor cells

The immune response against cancer cells often functions as a cycle involving many different tissues and organs. The immune response is often initiated in a tumor site by antigen-presenting cells that capture antigens released by cancer cells but then the activation of other immune cells (e.g., T and B cells) in the lymph nodes and trafficking through the nearby blood vessels is needed. Next, the activated immune cells must travel to the tumor site, where they kill tumor cells, releasing additional tumor antigens and reinitiating the cycle (Fig. 4). In this section, we first describe the antitumor immune response (i.e., the cancer-immunity cycle) as a framework for a discussion of the MPS literature and our perspectives for future work toward optimizing solid tumor immunotherapy. Tumors first arise from DNA mutations that lead to uncontrolled cell proliferation. These mutations also increase the synthesis of aberrant peptides and proteins, known as neoantigens, which are displayed on the tumor cell membrane or released upon cell death. In an optimal scenario, immune cells recognize the surface expression of neoantigens to pinpoint the presence of pathogens and foreign cells. These neoantigens are recognized by immune cells, triggering the antitumor immune response (Fig. 2a)^47^. Antigen-presenting cells (APCs), such as dendritic cells (DCs) and macrophages, recognize and phagocytose these peptides, which activates APCs. Activated APCs intravasate the lymphatic vasculature and migrate to the lymph nodes (Fig. 2b). Once APCs arrive in a lymph node, they present the peptides to effector cells (e.g., CD8 T cells) and regulatory immune cells (e.g., CD4 T cells). T cells then bind their T-cell receptors (TCRs), which subsequently scan the peptides presented by the APCs, resulting in T-cell activation. Activated T cells then intravasate into the lymph node afferent vessel and enter the bloodstream (Fig. 2c). Circulating immune cells extravasate from the vasculature and migrate to the tumor tissue guided by the inflammation, hypoxia, and necrosis caused by a tumor (Fig. 2a). Finally, effector immune cells (CD8 T cells and NK cells) recognize tumor cells and eliminate them via a variety of mechanisms, including secretion of perforins and granzymes or secretion of proapoptotic molecules (Apo2L/TRAIL). Tumor cell death (via necrosis or cytotoxic immune response) leads to the additional release of inflammatory factors and neoantigens, potentially amplifying the antitumor immune response^48–51^.

Although this cycle most often eliminates tumor cells before they form a detectable tumor, the tumor-immune cycle is a complex process orchestrated by multiple cell types (e.g., tumor cells, APCs, and T and NK cells) in different tissues, and alterations in any of the steps (e.g., immune exclusion, absence of inflammatory signals, or dysregulated vasculature) may compromise the capacity of the immune system to destroy a tumor^52^. This cycle might be compromised by a myriad of events: tumor antigens may not be detected by the immune system, the tumor may prevent immune cell penetration, and the TME may generate immunosuppressive conditions that hinder the effector response capacity of the immune system^48,51^.

Additionally, the selective pressure at a tumor site may promote the generation of additional mutations in cancer cells, which then contribute to a more heterogeneous tumor and a decrease in the number of neoantigens. The process leading to the elimination of neoantigen level in subsets of tumor cells (i.e., those capable of generating an immune response) is known as “cancer immunoediting”, and it makes cancer cells more likely to escape the cancer immune response^53–55^. Therefore, cancer immunotherapy is aimed to block tumor immune escape and reinitiate a self-sufficient cancer immunity cycle capable of an antitumor effector immune response^48^. This complex balance requires fine-tuning of immunotherapy interventions, which, as mentioned, presents important challenges to traditional in vitro modeling and creates opportunities for novel engineered in vitro models, such as microphysiological systems (MPSs). In the following sections, we introduce the concept and advantages of MPS in the field of immunology. We later review literature reports of MPSs used to recapitulate human tumor microenvironment and investigate the different stages of the cancer–immunity cycle.

## MPSs as an alternative for establishing better models of the cancer–immunity cycle

As mentioned in the previous section, solid tumors often contain many cell types, and microenvironmental cues are involved in the cancer–immunity cycle; this tumor heterogeneity and complex microenvironmental signaling are challenging to recreate in traditional 2D in vitro models. MPSs are alternatives to traditional in vitro models by offering better ability to recapitulate multiple key structural and environmental features of solid tumors. MPSs, and microfluidics approaches in general, are typically defined as systems operating at the microscale (i.e., confined to spaces smaller than 1 mm in one dimension)^56^, and they exploit physical properties that are common to the microscale, such as highly predictable fluid behavior (i.e., laminar flow)^57^. Another related advantage of MPSs over other in vitro culture setups is a consequence of the small volumes required by this set of technologies, making MPSs particularly useful for analyses with small samples, such as tumor biopsy samples^58^.

MPSs create environments with spatial dimensions closer to what occurs during cell‒cell and cell-tissue interactions. Specifically, the small dimensional space leads to less dilution of secreted factors (i.e., microliters vs. milliliters range in conventional in vitro platforms such as Transwell systems), facilitating cell‒cell signaling and the generation of biological gradients (e.g., chemotaxis gradients)^56,59^.

**Fig. 3:**
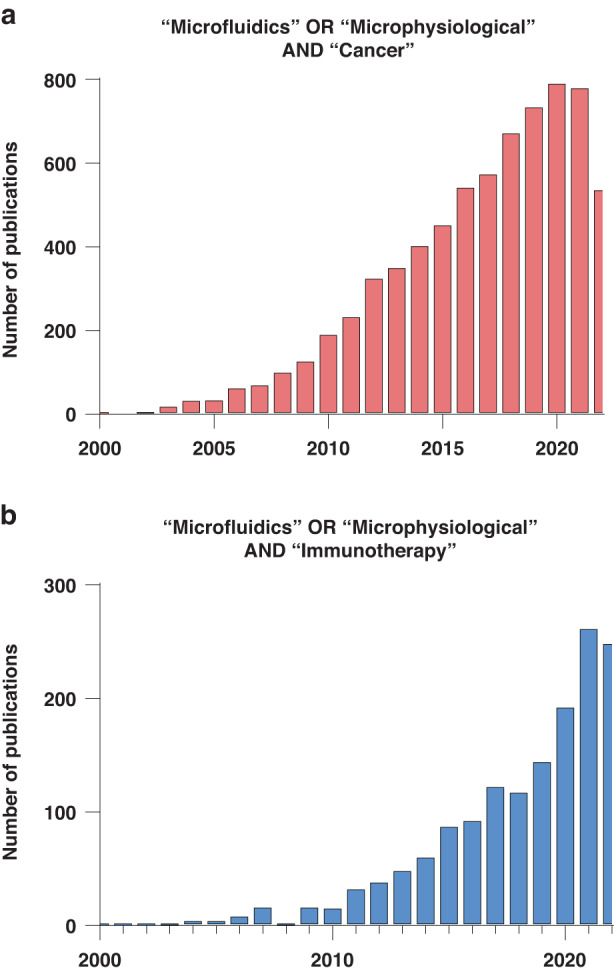
Publication trends in microfluidics for cancer and immunotherapy applications. Publishing trends for articles when “immunotherapy AND microfluidics” **b** and “cancer AND microfluidics” **a** were used as the search terms. PUBMED was used to generate the graphs on 8/21/2023 with the search terms as described in each graph title. The graphs reflect only publications after 2000 to ensure visual clarity

Fluid flow predictability has led to the use of MPSs to generate systems with fluid or hydrogel–fluid barriers^60,61^, allowing the generation of compartments within these devices. These compartments can be leveraged for generating individual cell culture chambers, where size, shape, cell composition and cell‒cell interactions can be engineered to suit experimental needs and mimic tissue architecture. It is from this capacity to mimic tissue and organ physiology that the concepts of organ-on-a-chip and microphysiological systems (MPSs) were derived. MPSs can be defined as microscale in vitro platforms that rely on the use of three-dimensional (3D) environments (e.g., multicellular spheroids), 3D matrices (e.g., collagen), and/or the culture of one or multiple cell types (e.g., tumor cells) to mimic specific features of in vivo organ physiology. Due to their appeal, the number of studies using MPSs has steadily increased in the past few years, with recent reports exploring their potential applications in the field of immunotherapy (Fig. 3).

All the advantages described in this section have made MPSs valuable tools in the study of the TME and immunotherapy. Microfluidics allows the generation of compartmentalized tissue culture systems in which multiple cell populations (e.g., tumor and stromal cells) are arranged to mimic the in vivo spatial organization of tumors. The models created using these platforms (i.e., MPSs) can include additional structures, such as vasculature, to study the role of these structures in cancer progression and immunosurveillance (e.g., tumor-induced angiogenesis, immune extravasation). Thus, MPSs are unique tools for exploring and accelerating the development of new immunotherapy treatments^62–64^.

## Opportunities and challenges of bioengineered in vitro models in solid tumor immunotherapy

**Fig. 4:**
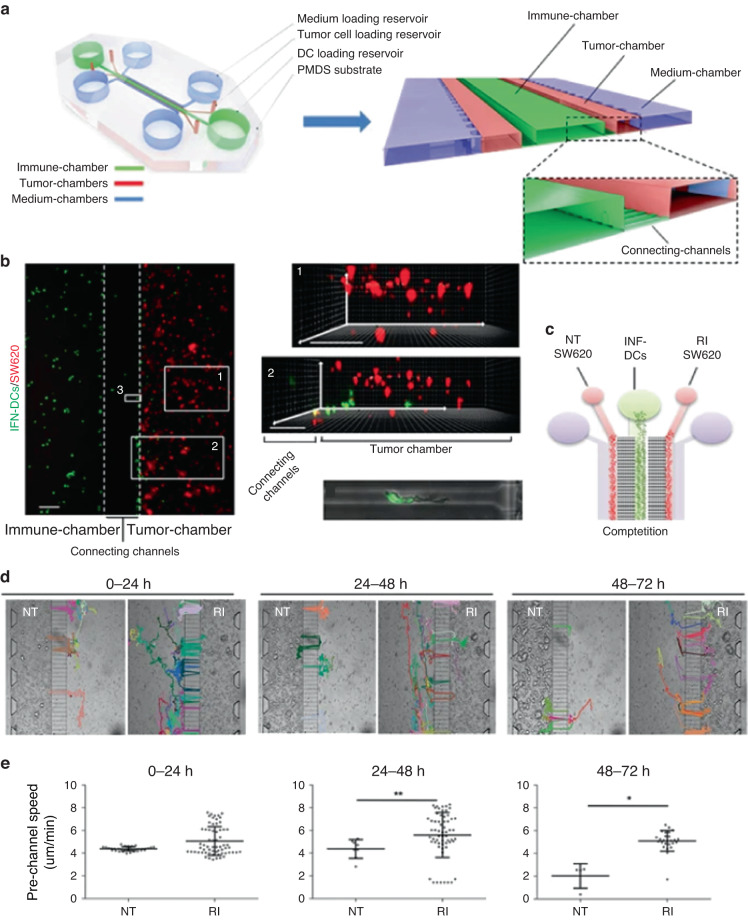
Evaluating immunotherapy efficacy by tracking dendritic cell (DC) migration toward tumor cells in a microfluidic device. **a** Schematic model showing the 3D microfluidic device used for real-time monitoring of DC migration toward cancer cells. The device consists of a central immune chamber with rounded loading reservoirs at both ends for DC loading connected through a network of narrow microchannels. An enlarged 3D section of the device is shown on the right. The boxed area represents the magnified view of the connecting channels. **b** DCs recognized colon cancer cells in the tumor microenvironment, took up antigens and migrated to the lymph nodes to activate T cells. **c** Schematic representation of one of the experimental setups used in the study: nontreated (NT) DCs vs. DCs treated with interferon-alpha and the histone deacetylase inhibitor romidepsin (treatment condition = RI). **d** Migratory patterns of DCs toward untreated (NT) and treated (RI) cancer in 72 h time-lapse experiments. **e** Scatter plots showing the migratory speed of IFN-DCs in the immune chamber (prechannel speed) and in the tumor chambers (postchannel speed) toward both NT- and RI-treated SW620 cells. Adapted from Parlato et al.^74^

The past few decades have seen advances in microfluidics, and microfabrication technologies have led to the development of engineered in vitro platforms that allow researchers to capture the tissue microstructure in advanced models. MPSs have been developed to mimic several steps of the cancer–immunity cycle, offering a new approach to studying cancer immunology. In this section, we provide an overview of microfluidic models developed to study the cancer–immunity cycle (Fig. 4).

## Inflammation and necrosis inside solid tumors

Inflammation and tumor necrosis are known drivers of malignant tumor progression and exert different effects on immunity through the different stages of the cancer-immunity cycle^65^. The initial inflammatory response is triggered by factors from dying cells at a tumor site (damage-associated molecular patterns, DAMPs) and signaling from the hypoxic tumor core, leading to tumor necrosis. These cues lead to the recruitment of monocytes and lymphocytes to a tumor site and induce the activation of these cells. This response also activates DCs, increasing their ability to take up antigens and present them to naive T cells^49,65^. In contrast, chronic inflammation may lead to an immunosuppressive environment that inhibits the antitumor response^66^. Hence, these mechanisms are intricately regulated and dependent on many cues from a tumor site and immune cell interactions among themselves and with tumor cells.

Hence, traditional 2D systems fail to recapitulate inflammatory stimuli, necrotic signaling, and immune cell migration into a tumor^67,68^. In terms of immune cell migration, immune cell extravasation, tumor penetration, and subsequent migration to lymph nodes have been areas of interest conducive to study with microfluidic models. MPSs have enabled the establishment of models that enable direct observation of the responses of DCs and macrophages to tissues undergoing inflammation and necrosis; that is, in MPS-generated models, immune cells migrate toward and penetrate the tumor mass, capture tumor neoantigens, and transition to an activated phenotype^69,70^. An example was reported by Um et al., who generated networks of interconnected microchannels to generate a microfluidic maze^71^. Immature DCs were loaded into the maze, and their migration toward a tumor or nonmalignant cells was captured by fluorescence microscopy, which also enabled monitoring of tumor cell–immune cell crosstalk and immune cell recruitment. The authors found that breast cancer cells (i.e., β-MEKDD 116) increased the number and speed of DCs successfully navigating the maze toward them compared to the number and speed of immune cells moving toward nonmalignant breast epithelial cells (i.e., Eph4 cells) and immune cells in a model without cancer cells. Notably, immature DCs navigated the maze through the shortest route, highlighting the capacity of immature DCs to detect malignant cells via chemotaxis. Molecular analysis revealed that β-MEKDD 116 breast cancer cells increased the secretion of growth arrest-specific 6 (Gas6), a signaling molecule known to induce growth arrest in its target cells (e.g., fibroblasts). The authors then used blocking antibodies to demonstrate that Gas6 was responsible for the increase in the speed and directionality of immature DCs in the maze. Finally, the authors found that DCs reaching the end of the maze expressed higher levels of mature DC markers than immature DCs treated with conditioned medium from β-MEKDD 116 or Eph4 cells, pointing toward the importance of migration and recruitment in mounting an antitumor immune response. Chernyavska et al. used an MPS with HEK293 cancer cells in a 3D hydrogel to study macrophage-based immunotherapy. They perfused M1- or M2-polarized macrophages through the MPS to study the rate of macrophage phagocytosis of cancer cells. They evaluated several antibody formulations targeting EGFR (expressed on tumor cells) to redirect and increase the macrophage phagocytosis rate by a process known as antibody-dependent cell phagocytosis^72^. Kim et al. used an MPS that included tumor cells and macrophages in a 3D hydrogel flanked by channels lined with endothelial cells. Their study demonstrated that macrophages promoted tumor cell invasion and metastasis by creating “microtracks” through the ECM and damaging cell‒cell junctions between endothelial cells, which promoted tumor cell invasion of the blood vessel surrogate^73^.

Parlato et al.^74^ used a microdevice comprising multiple chambers connected by a series of narrow microchannels to investigate the antitumor effector mechanisms of interferon-conditioned DCs (Fig. 4a). To this end, the authors cultured metastatic colon cancer cells (i.e., SW620 cells) embedded in a 3D hydrogel in one of the chambers, whereas primary DCs were perfused through the adjacent chamber (Fig. 4b, c). This study revealed that costimulatory signals were necessary for triggering the efficient migration of DCs. Treatment of cancer cells with immunotherapeutic interferon-ɑ and the histone deacetylase inhibitor romidepsin (abbreviated as RI) increased the number of DCs migrating into the SW620-containing chamber by twofold within 48–72 h of treatment (Fig. 4d, e). Molecular analysis revealed that this proinflammatory signaling increased tumor cell secretion of CXCL12 as well as DC expression of CXCR4 (i.e., CXCL12 receptor), indicating a role for this pathway in the results observed. Additional experiments using CXCR4 inhibitors demonstrated that the CXCL12-CXCR4 axis drove DC migration. Furthermore, the study revealed that RI treatment increased DC uptake of neoantigens and increased antitumor competence^74^.

**Fig. 5:**
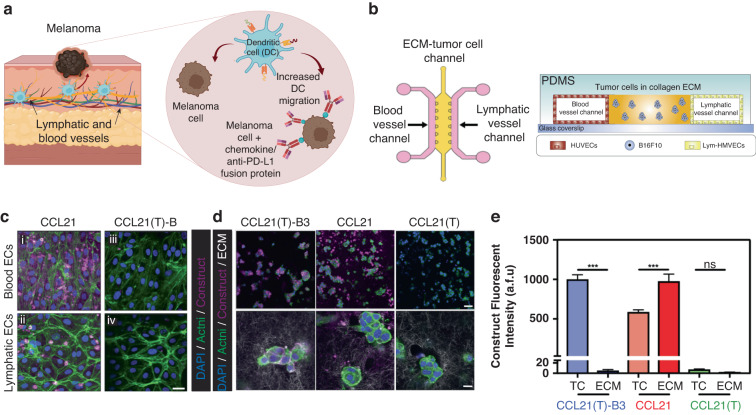
Evaluating the effectiveness and microenvironmental effects of new immunotherapies using MPS. **a** To optimize the response to immune checkpoint blockade by guaranteeing the presence of effector cells in the tumor microenvironment, a chemokine delivery system was designed. It consisted of a PD-L1-blocking single-domain antibody fragment and a charge-engineered chemokine CCL21 that promoted leukocyte trafficking into the TME. **b** Schematic showing the microfluidic device simulating the tumor microenvironment used to study immune checkpoint blockade therapy targeting. **c** The location of several structures in the tumor cell-extracellular matrix chamber. CCL21(T)-B3 was found in B16 melanoma cells. CCL21 bound to collagen I ECMs more efficiently, and CCL21(T) did not bind to cells or the ECM. **d** CCL(T)-B3 bound to tumor cells with higher selectivity than CCL21 or CCL21(T). **e** Quantification of the images in (**d**) showing that CCL21(T)–B3 selectively binds to tumor cells (TC) compared to CCL21 or CCL21(T). Adapted from Fang et al.^75^

Fang et al.^75^ used similar methods in their study, where they developed a chemokine/anti-PD-L1 nanobody fusion protein that simultaneously targeted exhausted immune cells and exclusion immune cells (i.e., immune cells close to the tumor but few that directly contact tumor cells)^76^. The authors fused a PD-L1-blocking single-domain antibody fragment to an engineered chemokine CCL21 molecule and evaluated the effects of this fusion protein binding to melanoma cells on DC migration (Fig. 5a). Next, the authors cultured B16-F10 melanoma cells in a 3D collagen hydrogel within an established microfluidic device^77^ (Fig. 5b). The left and right hydrogel flanks were lined with endothelial and lymphatic endothelial cells, mimicking blood and lymphatic vessels (Fig. 5c). Microscopy analysis revealed that targeting PD-L1^+^ tumor cells with the chemokine/anti-PD-L1 antibody increased the migration rate of DCs toward the tumor cells (abbreviated TC in the figure) compared to the rate of control cells (Fig. 5e). This study illustrated the potential of using an MPS to evaluate the efficacy of strategies to increase the recruitment of effector cells and, in turn, increase antitumor immune responses. The authors provided an effective proof-of-concept of MPS models contribution to advance our understanding of DC and macrophage migration toward tumor tissue and enhance the initial steps of the immune response to cancer.

## Migration to lymph nodes, antigen presentation, and immune activation

**Fig. 6:**
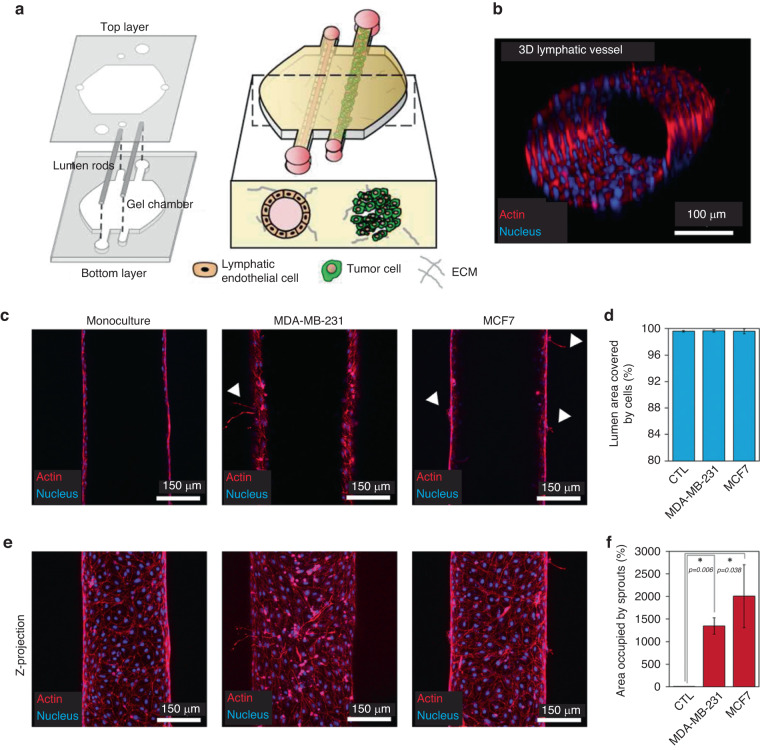
Lymphatic vessel migration and conditioning by solid tumors. **a** Schematic representation showing the microfluidic platform used to evaluate how the presence of a solid tumor modifies the structure and functionality of a lymphatic vessel. **b** Confocal microscopy images of lymphatic vessels in monoculture and coculture consisting of MCD7 and MDA-MB-231 cells (upper row) and *Z*-projected images (lower row). **c** Quantification of cell coverage for each culture condition showing an average percentage of cell coverage >99% for all conditions. **d** Crosstalk with breast cancer cells induced lymphangiogenic sprouting in the vessels. **e** Images show the maximum intensity *Z*-projection of the lumens shown in (**d**). **f** Bar graph shows the quantification of the hydrogel area occupied by lymphangiogenic sprouts observed in the *Z*-projections shown in (**e**) in monoculture conditions as well as co-culture with MDA-MB231- and MCF7 cells. Adapted from Ayuso et al.^83^

After APCs (e.g., DCs) take up an antigen, they intravasate into surrounding lymphatic vasculature and migrate to tumor draining lymph nodes^78^ in a process requiring multiple intravasation and extravasation steps. In the lymph node, antigens are presented to activate T and B cells and, in turn, mount an effective antitumor immune response. The diversity of tissues and 3D structures (e.g., vessels, lymph nodes) presents considerable obstacles to investigate these steps in the tumor immunity cycle in vitro using traditional systems. However, microfluidics excels at providing high structural control over cell culture systems, which presents an opportune application for an MPS^79^. The MPS mimicking of blood vessels and in vitro vascular networks has been well established^61,80–82^. Therefore, recently, researchers have leveraged this technology to investigate tumor conditioning through blood and lymphatic vessels, thereby regulating immune cell transmigration prior to their activation and their antitumor activity. For example, as reported by Ayuso et al.^83^, who investigated the effects of nearby tumor cells on 3D blood and lymphatic vessels, 3D tubular vessel models were developed with a well-established mold-casting method^84^. Briefly, this method consists of polymerizing a collagen mixture in a microfluidic device around a PDMS rod^85^ (Fig. 6a). After collagen polymerization, the PDMS rod is removed to generate an empty tubular structure through the collagen hydrogel, which is lined with lymphatic endothelial cells to create a confluent monolayer (Fig. 6b). This approach allowed the authors to compare the effects of tumor conditioning on blood and lymphatic vasculature (Fig. 6c), revealing differences in nutrient and protein permeability and chemokine secretion when a tumor was present (Fig. 6d). This approach was also used to evaluate the different effects of conditioning when different tumor types were used (e.g., breast and head and neck cancers)^86^. Future studies should focus on investigating the ability of immune cells in these models to monitor immune cell transmigration to optimize the early steps of the cancer–immunity cycle.

**Fig. 7:**
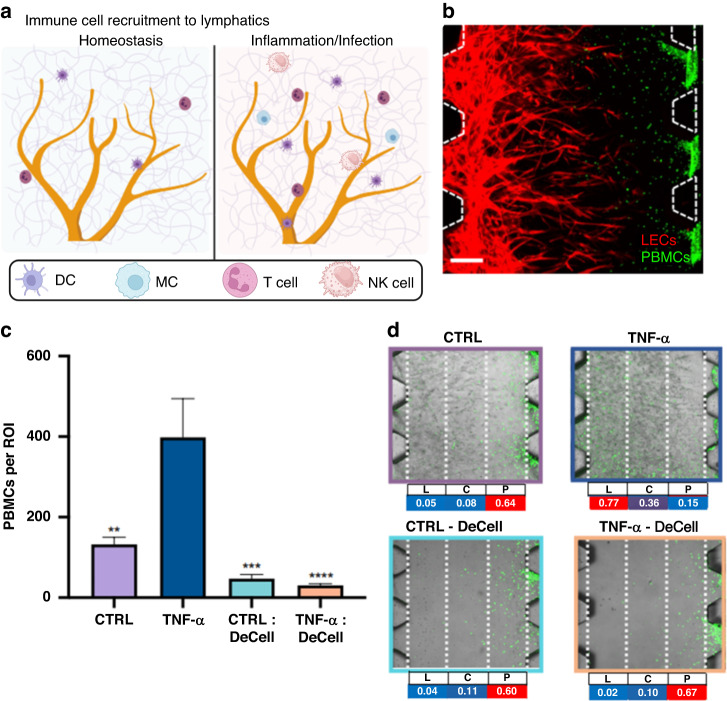
Recapitulating pathological immune cell recruitment by engineered lymphatics. **a** Immune surveillance and recruitment in the lymphatic milieu depicted schematically according to tissue condition. **b** PBMC (green) infiltration assay through a hydraulic pressure difference gradient. **c** Quantitative analysis of PBMC infiltration assay in high-flow lymphatics stimulated with TNF-α and/or decellularized. **d** Representative images and quantitative heatmap analysis of PBMC distribution (green) within the gel region for each experimental condition. Adapted from Serrano et al.^90^

A subsequent step required in the immune activation cascade is immune cell recruitment. Specifically, cell migration studies have traditionally benefited from microfluidic technologies^87,88^. Thus, a recent study adapted a well-characterized microfluidic device^89^ to simulate the lymphatic response to inflammation (local stromal cells release tumor necrosis factor alpha (TNF-α), transforming growth factor-β (TGF-β), and interleukins), which leads to immune cell recruitment to tumors or infection sites through chemotactic signaling in vivo^90^. The authors used this device to generate an MPS and investigate the role of interstitial flow in the recruitment of immune cells to lymphatic vessels (Fig. 7a). Human peripheral blood mononuclear cells (PBMCs) were perfused through the device using a gravity-driven flow system (discussed in more detail in subsequent sections) and monitored through confocal microscopy (Fig. 7b). The authors observed higher rates of migration and extravasation into the lymphatic vasculature under inflammatory conditions (TNF-α) compared to control conditions (Fig. 7c, d).

The studies by Ayuso and Serrano^83,90^ discussed in this section focused on investigating the role of lymphatic vasculature in the antitumor immune response. Their systems allowed the investigation of specific growth factors and chemokines and their roles in barrier function and interstitial fluid flow, respectively, as both proteins types play roles in the drainage rate of soluble factors. However, more research is required to better understand the mechanisms governing lymphatic regulation of the antitumor immune response and potential clinical opportunities stemming from this understanding.

Following DC capture of tumor antigens, chemotactic gradients guide DCs, which intravasate, reach the lymph node, and interact with T cells. The chemotactic gradients guiding DC navigation are not completely understood, and microfluidic tools, which have demonstrated their potential in generating stable and controlled molecular gradients, are opportune tools to investigate their role in cellular response (e.g., endothelial cell angiogenesis guided by VEGF)^91,92^. An example of this application was reported by Koria et al.^93^. The authors described a microdevice including two parallel microchannels connected by a series of narrow capillaries and that was constricted in the center (i.e., “H” design). DCs were perfused through one of the lateral microchannels while the outlet of the same channel remained closed, forcing the cells to flow through the series of central capillaries. Single DCs were captured in the central microchannels due to the channel constriction at the center. Next, the authors perfused chemotactic signals (e.g., CCL19) through one of the lateral microchannels to generate chemotactic gradients across the center of the device. DC migration in response to these gradients revealed two different DC subpopulations. Despite their comparable size, one of these DC subpopulations exhibited a 5-fold faster migration rate, suggesting multiple phenotypes of activated DCs^93^. Future studies can use a similar approach to identify new therapies that promote DC migration toward the lymph nodes, which in turn may enhance the adaptive and, potentially, the innate immune response.

In vivo, after reaching a lymph node, immature DCs acquire a mature phenotype characterized by a higher surface expression of MHC molecules and expression of stimulatory cytokines such as IL-2 and surface ligands such as B-7. Mature DCs can present captured antigens to T cells in the lymph node via MHC class-II (HLA-II in humans)—TCR interactions (on the T-cell surface). Provided that the TCR and the presented antigen are complementary, the bound T-cell is activated and then proliferates and acquires other effector functions (e.g., CD8 T cells become cytotoxic cells, and CD4 T cells become helper T cells), thereby initiating the adaptive immune response. Efficient activation of the adaptive immune response depends on the recognition of tumor antigens by highly specific clones of T cells expressing a complementary TCR. Additionally, the number of tumor antigens generated by a specific tumor varies considerably among different patients, which is consistent with the large interpatient heterogeneity observed in the clinic. Such high heterogeneity indicates that single-cell approaches are needed to better appreciate the variability and effectiveness of the trigger an immune response.

**Fig. 8:**
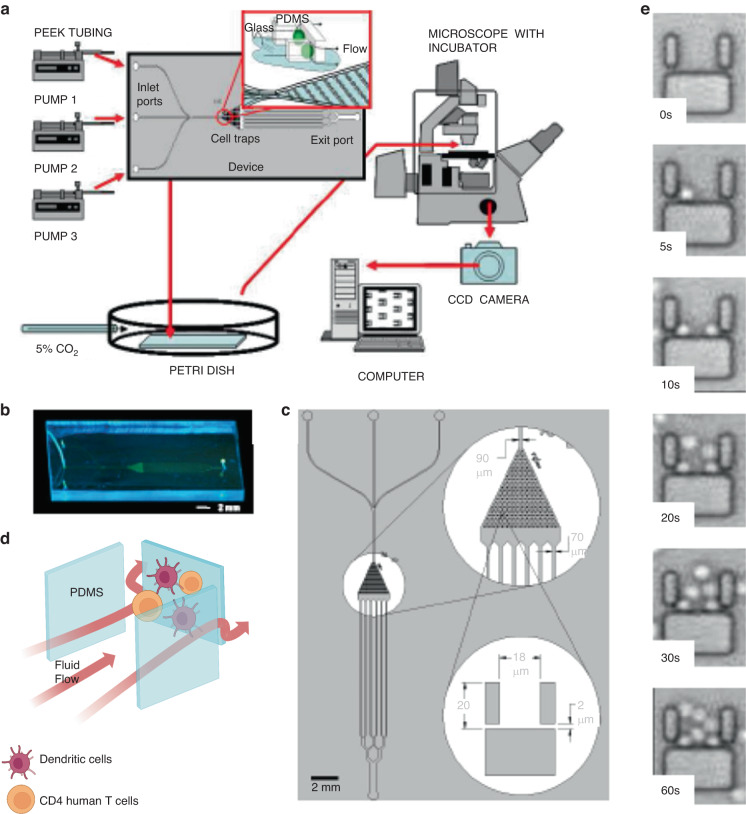
Microfluidic multitrap experimental setup to study T-cell activation by DCs. The immunological synapse is key to a fundamental understanding of a successful adaptive immune response. Studying T-cell–APC interactions in vitro is challenging, however, due to the difficulty of tracking individual, nonadherent cell pairs over time. **a** Schematic illustration of the experimental setup for the cell trap device. **b** Photograph of the device after the PDMS structure is bonded to a glass coverslip. **c** A schematic overview of the device proposed by Foley et al.^95^. **d** Close-up schematic of the cell trap region showing the design and arrangement of individual cell traps. **e** Time-lapse imaging of a single representative cell trap within the microfluidic device, demonstrating that traps are loaded with cells within minutes of their introduction

Given their capacity to manipulate small volumes, microfluidic technologies are well suited for single-cell analysis applications. An example was presented by Faley et al.^94^. The authors designed a microdevice with a large array of microfluidic traps to study T-cell activation by DCs (Fig. 8a–c). The traps were able to retain single cells, so the authors were able to track individual cell behavior (Fig. 8d). The authors perfused human naive T cells through the microdevice and then stimulated them with known activation signals such as chemicals (CaCl_2_, ionomycin), chemokines (e.g., IL-2), or antibodies (anti-CD3 and anti-CD28 antibodies), demonstrating the capacity of the device to monitor naive T-cell activation, observed as intracellular changes in calcium flux at the single-cell level. Additionally, the authors exposed DCs to lipopolysaccharides (i.e., molecules commonly present in gram-negative bacteria that trigger an immune response) to induce dendritic cell maturation. Mature DCs induced naive T-cell activation, and the authors used the single-cell analysis capacity of the device to identify clones with more robust and time-persistent activation (Fig. 8e). Although this approach was limited to bacterial antigens, future studies could leverage similar platforms with specific tumor antigens to (1) evaluate whether a specific patient would respond to tumor antigens and (2) identify and isolate those T-cell clones with the strongest antitumor response for downstream expansion^95^.

Given the importance of antigen presentation in initiating the adaptive immune response, many researchers have sought to capitalize on this step of the adaptive immune response to increase antitumor immunity. One such approach (i.e., cancer vaccines) consists of isolating APCs ex vivo and loading tumor antigens in vitro to educate immune effector cells. Antigen-loaded APCs are later injected into the patient’s bloodstream, where they migrate to lymph nodes and activate a tumor antigen-specific T-cell response. A similar approach involves presenting antigens to B cells to educate them and generate a humoral immune response. Given their abundance in the bloodstream and their migratory potential to lymphoid tissue B cells are an interesting choice for the development of cell-based cancer vaccines. However, effective antigen loading in vitro is still difficult, as poor antigen capture by an APC, inefficient antigen processing, and failure of MHC-I/II receptors to present antigens are all potential pitfalls. Therefore, despite extensive research, only one APC-based vaccine has been approved by the FDA thus far^96^. Therefore, alternative approaches to antigen loading are being developed to deliver macromolecules of choice and circumvent the aforementioned issues.

**Fig. 9:**
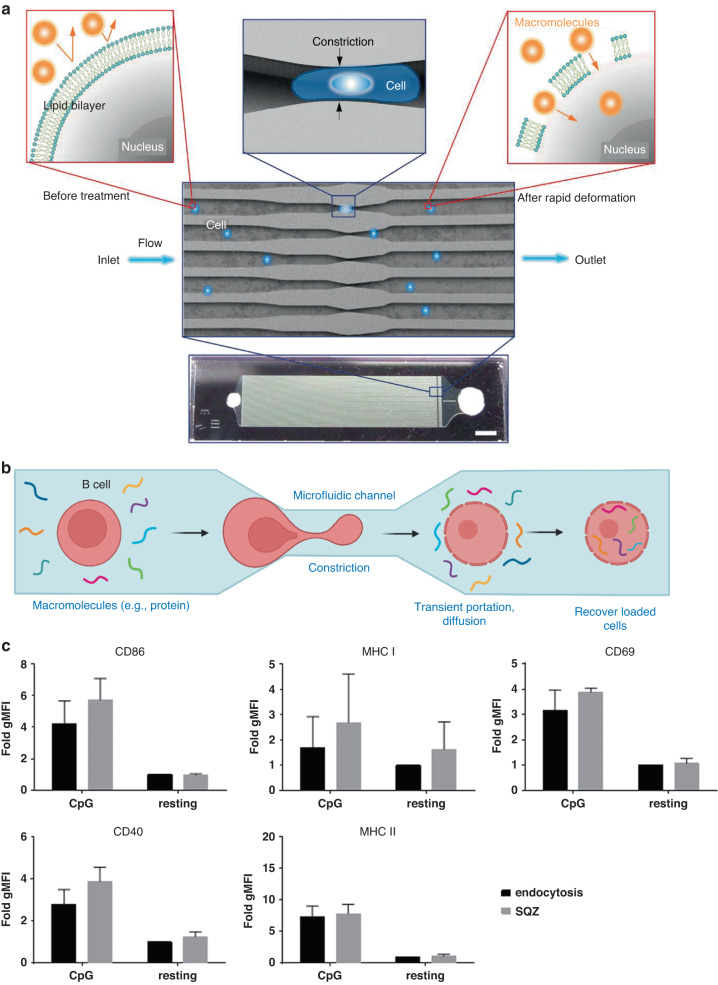
Microfluidic squeezing for macromolecule delivery to B cells. **a** Schematic depiction of the macromolecule delivery strategy for B cells and image of the device used by the authors. The rapid deformation of a cell as it passes through a microfluidic constriction generates transient membrane holes. **b** B-cell activation is not altered by cell squeezing. Quantitative analysis of activation and antigen-presentation marker expression after 48 h. **c** Quantitative analysis of activation and antigen-presentation marker expression after 48 h in media alone (resting) or CpG B 1826 stimulation (CpG), calculated as fold change in geometric mean fluorescence intensity above resting, endocytosis cells. Adapted from Szeto et al., Sharei et al., Shinde et al.^98^^,148^^,^^153^

One such alternative approach, which has shown promising results, is microfluidic mechanoporation^97^. With this approach in mind, Szeto et al. fabricated a microdevice including a series of parallel microchannels with one or multiple 6 μm constrictions^98^ (Fig. 9a). Flowing cells (e.g., DCs, B cells) through the constrictions created transient pores in the cell membrane, allowing macromolecules such as 40 kDa dextran to be delivered to cytoplasm (Fig. 9b). The authors explored different designs by changing the number of constrictions and their lengths, thereby identifying the optimal parameters to ensure maximum delivery and cell viability. Subsequent experiments demonstrated that complete proteins and antigens delivered by mechanoporation to APCs were correctly processed and presented by MHC class I molecules. In vitro, APCs subjected to mechanoporation-based antigen delivery successfully primed and activated antigen-specific CD8^+^ T cells, in turn increasing the expression of granzyme B, TNF-α, IFN-γ, IL-2, or CD137 (Fig. 9c). Further experiments in vivo demonstrated that after mechanoporation, APCs induced antigen-specific T-cell activation and proliferation in the mouse spleen^98^. This success was also achieved with genetically engineered T and NK cells, especially chimeric antigen receptors^99–102^. Although these techniques are still in their proof-of-concept stage, in time, they may contribute to the accelerated implementation of engineered immune cells as clinical therapeutics.

## Immune cell recruitment into solid tumors

**Fig. 10:**
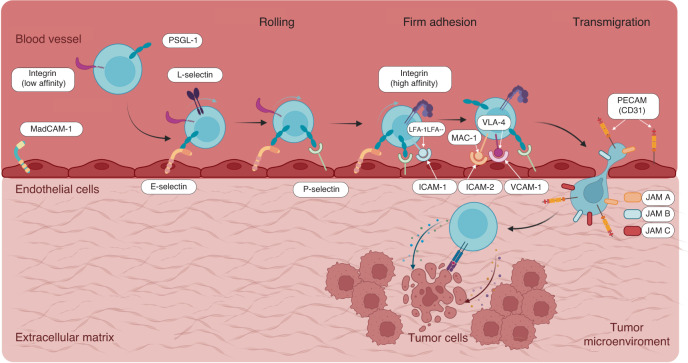
Model of the leukocyte adhesion cascade and immune cell infiltration into the tumor microenvironment. Adapted from Chae et al.^107^ A step-by-step process involving leukocyte rolling, tight adhesion, and transmigration into the circulatory system and movement toward a tumor site. This cascade is mediated by various molecules interacting with each other, as shown in the schematic. Abbreviations: PSGL-1: P-selectin glycoprotein ligand-1, MAdCAM-1: mucosal vascular addressing cell adhesion molecule 1, LFA-1: lymphocyte function-associated antigen 1, Mac-1: macrophage-1 antigen, VLA-4: very late antigen-4, ICAM-1/2: intercellular adhesion molecule 1/2, VCAM-1: vascular cell adhesion molecule-1, PECAM: platelet endothelial cell adhesion molecule, JAM A/B/C: junctional adhesion molecule A/B/C

Despite the promising outlook offered by immunotherapy, solid tumors have a variety of mechanisms to enable their cells to evade antitumor immune surveillance. As discussed in the previous section, tumor cells can interfere in antigen presentation or in the activation of effector cells of the immune system (e.g., T cells, NK cells) to promote their own proliferation. Moreover, tumor cells can mitigate the antitumor response via the recruitment of activated immune cells (e.g., effector cells such as T and NK cells) to a tumor site. A successful immune cell recruitment process requires specific soluble factors and physical interactions between immune cells and surface mediators in the vasculature^103–105^. Briefly, once leukocytes are activated, they need to reach a tumor to exert their cytotoxic effects. DAMPs activate the innate immune system by interacting with the pattern recognition receptors (PRRs) that sense DAMPs^106^. DAMP recognition by innate immune cells in the tumor site (e.g., resident macrophages) promotes the generation of cytokines (e.g., IL-1), chemokines, and other proinflammatory mediators that orchestrate the recruitment of leukocytes from the blood to the affected tissue. The secretion of IL-1, TNF-α, and C5a triggers blood vessel endothelial cells to express cell adhesion molecules (e.g., E-selectin and P-selectin) on the cell membrane^107^. Leukocytes interact with the vasculature via a rolling movement (Fig. 10) mediated by the interaction between leukocyte surface proteins (e.g., LFA-1, Mac1, and VLA4) and endothelial cell surface proteins (e.g., ICAM-1, ICAM-2, and VCAM-1). Thus, a leukocyte binds tightly to the endothelium. Then, the leukocyte extravasates in a process called diapedesis, which is mediated by the surface receptor PECAM-1 (also called CD31) that is expressed on both leukocytes and vascular endothelial cells. Following this process, the leukocyte migrates through the basement membrane into the tumor-affected region^105,108^.

The tumor vasculature is generally immature, with a tortuous structure and hyperpermeable vessels, characterized by poor pericyte coverage and an abnormal basement membrane^109–111^. This poor architecture results in compromised structure and functionality, which tend to contribute to the increased delivery rate of nutrients and oxygen to a tumor^109,112–114^. In addition, these vascular traits may decrease the antitumor response and become an obstacle to immunotherapy^115^.

**Fig. 11:**
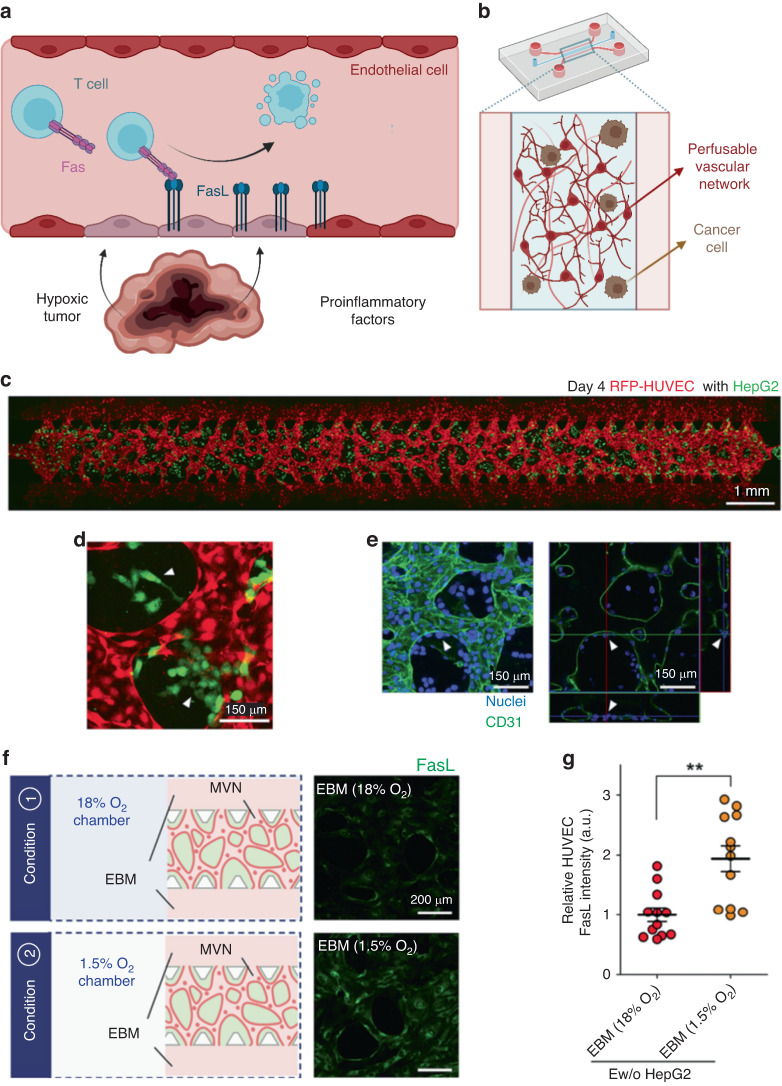
An MPS recapitulating tumor-induced endothelial FasL expression increase and T-cell apoptosis in the tumor microenvironment. **a** In vivo scenario recapitulated in an MPS: upregulation of endothelial FasL expression due to hypoxia and immunosuppressive factors of the TME induced apoptosis of cytotoxic T cells. **b** The schematic shows a constructed 3D MVN with tumor cells in a fibrin gel inside a microfluidic device. **c** On-chip microvascular network (MVN) surrounding tumor cells in a 3D fibrin gel and structural analysis results. RFP-HUVECs (red) MVN with HepG2 hepatocarcinoma cells (green) inside a microchannel. **d** Enhanced fluorescence microscopy image of the MVN. **e** CD31 (green) on the MVN remains intact when HepG2 tumor cells (arrows) are attached to the extraluminal region of the MVN. Cell nuclei (blue) are stained with Hoechst 33342. **f** MVNs were treated with EBM and placed in chambers with 18% O_2_ or 1.5% O_2_ for 20 h. **g** Images show immunofluorescence staining for FasL (green) expressed on the MVN with 18% O_2_ or 1.5% O_2_. Adapted from Kim et al.^116^

One of the factors affecting the success rate of immunotherapy is the ability of immune cells to reach a solid tumor. Previous studies have shown that solid tumors hijack the surrounding vasculature to render it less immunogenic by suppressing immune cell recruitment and penetration. Kim et al.^116^ evaluated the expression of Fas ligand (FasL) in tumor-associated endothelial cells. FasL binds to its receptor (Fas, CD95) on a target cell membrane to promote homeostatic T-cell death, preventing cytotoxic antitumor activity (Fig. 11a)^117^. It has been reported that TME-specific hallmarks such as hypoxia, reactive oxygen species (ROS) and tumor-derived cytokines contribute to the upregulation of FasL on tumor endothelial cells^118,119^. Among the many microfluidics-based studies recreating the TME, a recent study capitalized on this body of research to develop an MPS of immune cell recruitment and tumor immunosuppression. The authors used a well-characterized microfluidic device to generate a 3D liver tumor and vasculature MPS to investigate the mechanisms of tumor immunosuppression (Fig. 11b).

Endothelial cells (HUVECs) were embedded in a 3D fibrin hydrogel with tumor cells (HepG2) in a PDMS microfluidic chip to recreate the effects of the tumor on a mature tumor microvascular network (MVN) (Fig. 11c–e). Hypoxia (hypoxic chamber at 1.5% O_2_) (Fig. 11f) triggered the overexpression of FasL on endothelial cells in the MVN. Next, cytotoxic T cells (Jurkat cells) were perfused. Studies with this MPS revealed that hypoxia and tumor inflammation, in turn, increased the rates of apoptosis of cytotoxic T cells (via Fas-FasL interactions)^28^ (Fig. 11g). The authors also identified a cocktail of cytokines that were upregulated under hypoxic conditions and may be responsible for triggering FasL overexpression: CXCL7, CXCL 1-2-3, TGF-β2, CCL4, and LIF. Finally, the authors determined that this immunosuppressive mechanism mediated by overexpressed FasL was inhibited by FasL-Fas inhibitors.

**Fig. 12:**
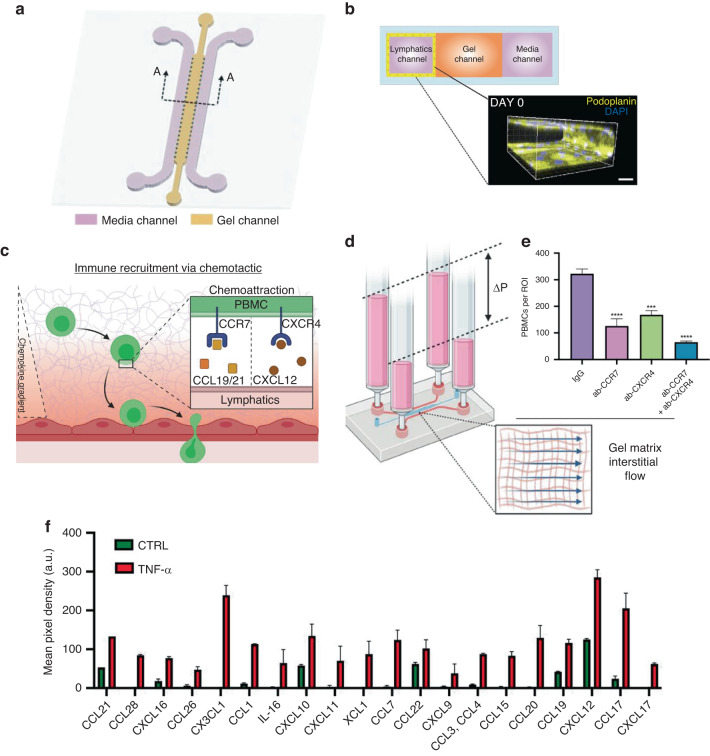
Microfluidic system mimicking human lymphatic microvasculature for physio-/pathological transport studies. **a** Schematic of the microfluidic device with the corresponding channels labeled. **b** Cross-section view of the microfluidic device used by the authors. Confocal reconstruction of a lymphatic monolayer seeded in the medium channel with podoplanin staining of the cell membrane and DAPI staining of nuclei. Scale bar = 100 μm. **c** Schematic showing the in vivo scenario recapitulated in the MPS model. During an inflammatory response, chemotactic signaling axes recruited immune cells via concentration gradients of the respective chemokines. **d** Schematic representation of the interstitial flow setup, where a hydrostatic pressure difference (ΔP) drove interstitial flow across the gel matrix compartment. **e** Quantitative analysis of PBMCs infiltrated via engineered high-flow lymphatics stimulated with TNF-α and under corresponding conditions of PBMCs reconstituted with neutralizing antibodies or IgG isotypes. **f** Quantitative analysis of cytokine secretion from cells in lymphatics established with interstitial flow and one experimental group preconditioned with TNF-α. Adapted from Serrano et al.^90^

Serrano et al.^90^ further investigated the role of cytokines as homing molecules in the process of immune recruitment in their MPS. Specifically, they focused on well-established chemotactic pathways known to drive immune cell recruitment and homing during inflammation: (1) CCL21 and CCL19, agonists of the CCR7 receptor in immune cells, which migrate toward the lymphatic endothelium for extravasation and (2) stromal and endothelial cell-secreted CXCL12, agonists of the CXCR4 receptor in immune cells, respectively (Fig. 12c). They leveraged an MPS with a 3D lymphatic vessel network and PBMCs to evaluate the role of these pathways in PBMC recruitment in their system (see Fig. 12a to see the device and Fig. 12b to see the model), where they included interstitial flow (Fig. 12d) and evaluated immune cell recruitment, which is reported as the number of PBMCs per region of interest (Fig. 12e). The authors also evaluated the changes in autocrine secretion from lymphatic vessel cells after treatment with the proinflammatory factor TNFα (Fig. 12f). By performing iterative inhibitor experiments with their MPS, the team revealed a role for both pathways in immune cell recruitment to lymphatic vessels.

**Fig. 13:**
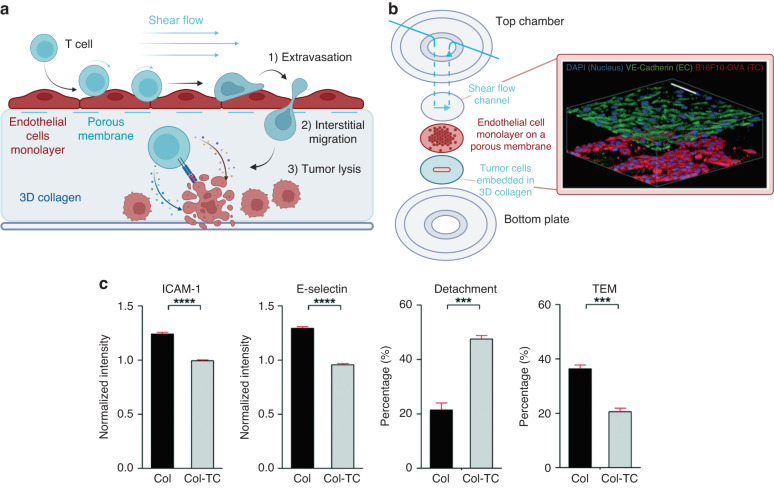
MPS used to investigate T-cell infiltration into solid tumor tissues. **a** Physiological process of T-cell infiltration within a solid tumor recapitulated with the microfluidic platform. **b** Schematic structure of the multilayered blood vessel/tumor tissue chip. An endothelial cell (EC) monolayer cultured on a porous membrane and a 3D collagen gel containing tumor cells (abbreviated as TCs) are located between the top chamber and the bottom plate. **c** Expression levels of the adhesion molecules ICAM-1 and E-selectin on endothelial cells in the presence (Col-TC) and absence (Col) of tumor cells in a collagen gel (height = 200 μm). Adapted from Lee et al.^120^

Following immune cell recruitment and activation in lymph nodes, immune cells in the blood stream reach the tumor site, and they extravasate through the blood vessels and infiltrate solid tumor tissues to exert their cytotoxic antitumor functions (Fig. 13a). Immune checkpoint blockade (ICB) therapy and CAR T-cell therapy are two recent examples of cancer immunotherapies based on the T-cell capacity to recognize and kill tumor cells, although these treatments are effective only for a small percentage of patients. The enhancement of T-cell infiltration into solid tumor tissues is one major problem in cancer immunotherapy since physical contact between tumors and T cells is required for effective antitumor T-cell cytotoxicity. Recent studies have reported the use of an MPS to study the late steps in antitumor immunity. One such study was reported by Lee et al.^120^, who fabricated a multilayered device to investigate tumor cell–blood vessel interactions and their impacts on T-cell extravasation and tumor killing (Fig. 13b). The MPS described by Lee comprised a top chamber, a porous membrane covered with an endothelial cell monolayer, and a collagen gel hydrogel containing tumor cells. T cells injected through the top fluidic chamber underwent dynamic interactions with endothelial cells, including intraluminal crawling and transendothelial migration. After extravasation, the T cells displayed directional migration toward tumor cells, demonstrating their capacity to detect the presence of tumor cells.

This MPS was also able to recapitulate other key functional effects of tumor cell–endothelial cell interactions. First, endothelial cells in the TME failed to activate in response to proinflammatory molecules (e.g., TNF-α). The authors observed a downregulation of adhesion molecules, such as intercellular adhesion molecule 1 (ICAM-1) and E-selectin (Fig. 13c), which was consistent with previous reports. Furthermore, more T cells interacting with endothelial cells under tumor coculture conditions (abbreviated Col-TC in Fig. 13c) exhibited detachment and reduced transendothelial migration rates than controls (Col) cells. The authors explained these observations as results of endothelial cell anergy caused by tumor cells. The cell anergy was reversed by an anti-VEGF drug, which also promoted T-cell infiltration, consistent with clinical observations. These results highlighted the utility of the presented model for preclinical immunotherapeutic evaluation and basic tumor immunology research^120^.

## Immune cell cytotoxicity inside solid tumors and the role of the tumor microenvironment

The high antitumor immunotoxicity of CD8^+^ T and NK cells has been established through numerous studies over the past few decades^121,122^. For this reason, most immunotherapies have focused on increasing NK and T-cell responses and minimizing tumor-derived immunosuppressive effects on these two immune cell types. Some important challenges to using cell suspensions and 2D assays for these studies include incomplete recapitulation of TME-induced immunosuppression and immune cell exclusion, which are known limiting factors of the antitumor immune response. This section explores studies aiming to investigate the factors regulating the effector function of T cells, CAR-T cells, and NK cells against tumor cells, as well as strategies aiming to increase their therapeutic efficacy.

## T-cell cytotoxicity

Early studies investigating T-cell cytotoxicity in MPSs leveraged PBMCs or T-cell lines to generate a proof of concept. Zboralski et al.^123^ generated heterotypic 3D organoids with cells from different tumor types (e.g., colon and brain cancer) and cancer-associated fibroblasts to evaluate the effect of CXCL-12 inhibition on the efficacy of T-cell PD-1 blockade. CXCL12 had previously been shown to exert a protective effect against checkpoint inhibitors through a mechanism involving T-cell exclusion. Thus, the authors sought to evaluate whether inhibiting CXCL12 action increases cancer cell sensitivity to PD-1 blockade. T-cell recruitment was evaluated in suspension by incubating PBMCs with tumor spheroids for 3 days, washing nonattached and loosely attached immune cells, and dissociating spheroids to evaluate the resulting cell populations via flow cytometry. PD-1–dependent T-cell activation was assessed after T-cell coculture with spheroids using a commercially available luminescence assay with a T-cell line (Jurkat cells). The results demonstrated that the use of the CXCL-12 inhibitor L-RNA-aptamer NOX-A12 increased T-cell infiltration in a dose-dependent manner in all the cell types analyzed. When combined with PD-1 blockers, the CXCL-12 inhibitor induced higher T-cell activation within the spheroid and decreased tumor volume than single-agent treatments. This study successfully showed that multitarget blockade may increase the efficacy of checkpoint inhibitors in 3D models and opened the door for similar applications of MPS technology to evaluate 3D T-cell migration. Further studies may also incorporate other relevant tissues or TME cell types to better understand the influences of the TME on immune exclusion and increase the translatability of these results.

A similar study was reported by Ritter et al.^124^, who also used a triple-channel device to demonstrate the role of the STING pathway in immune recruitment during triple-negative breast cancer (TBNC). Their MPS consisted of TBNC spheroids embedded in a collagen hydrogel in the central microchannel. The addition of ASU-S100, a STING agonist, led to increased secretion of chemoattract cytokines such as CXCL10, CCL5, and TNFβ. Subsequent perfusion of CXCR3^+^ T cells (Jurkat cells) through the lateral channels resulted in a chemotactic movement toward the tumor spheroids treated with ADU-S100. Given that in vivo solid tumors commonly exclude immune cells, this study showcases the potential of microfluidic devices to evaluate genetically engineered immune cells with increased chemotactic potential.

Kitajima et al.^125^ also leveraged the same device to explore the role of STING suppression associated with LKB1 in KRAS-driven lung cancer in immune escape. LKB1^−^/^−^ KRAS lung cancer is one of the most aggressive subtypes of lung cancer and responds poorly to ICIs. In a similar approach to the previous ones described, the authors generated LKB1^−^/^−^ KRAS -driven lung cancer cells and assembled them as spheroids. Next, they embedded these spheroids in a collagen hydrogel in the central microchannel, and then, they used the lateral channels to perfuse T cells (Jurkat). Their study demonstrated that loss of LKB1 led to STING downregulation, which in turn reduced the expression of multiple cytokines and chemokines involved in immune recruitment (e.g., CXCL13). Therefore, compared with control tumor spheroids, LKB1^−^/^−^ KRAS-driven tumor spheroids exhibited low levels of immune cell infiltration. Additionally, the authors demonstrated that LKB1 suppression led to the downregulation of PD-L1 expression on the cell surface. Interestingly, LKB1 restoration led to increased PD-L1 expression and immune cell recruitment. Overall, the researchers in this study used microfluidic devices to explore the molecular mechanisms behind LKB1^−^/^−^ KRAS-driven lung cancer in-depth, and the results may lead to much-needed therapies to treat aggressive lung cancer.

## CAR-T-cell cytotoxicity

As discussed, numerous genetic engineering strategies are being explored to generate more efficient immune cell formulations (e.g., CAT and CAR NK cells). Solid tumors impose immunological and physical constraints on the efficacy of CAR T-cell therapy, and these traits are challenging to recapitulate in traditional preclinical analysis with 2D cultures. A recent study generated MPSs with lung and breast cancer cells (A549 and MDA-MB-321 cells, respectively) to mimic the architectural and phenotypic characteristics of primary tumors and evaluate the anticancer activity of receptor tyrosine kinase-like orphan receptor 1-specific (ROR1-specific) CAR T cells.

**Fig. 14:**
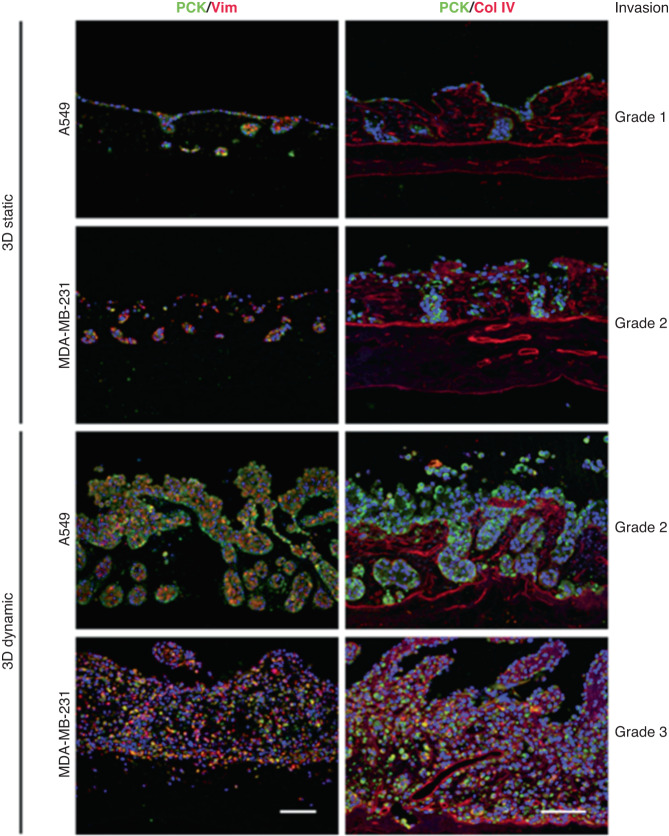
Recapitulating the effects of biophysical cues on lung and breast tumor microenvironments using microfluidic devices. Lung cancer and breast cancer cells were cultured under static and dynamic conditions. Tumor composition and architecture were evaluated by immunofluorescence techniques. Left column: pan-cytokeratin (PCK, shown in green) and vimentin (Vim, shown in red). Right column: Pan-citokeratin (PCK, green) and collagen IV (Col IV, red). Nuclei are counterstained with DAPI (blue), as shown in both columns. Adapted from Wallstabe et al.^127^

One of the main drawbacks of 3D in vitro models used to study immune cell infiltration and tumor cell killing is the use of simplistic hydrogels (e.g., collagen) that do not capture the structural or component complexity of the in vivo extracellular matrix^126^. The MPS described in a report of CAR-T-cell evaluation consisted of decellularized porcine scaffolds that were amenable to ex vivo medium perfusion (i.e., flow studies). The authors showed that differences in architecture and cell composition between static and dynamic (i.e., fluid flow) conditions were apparent after histological staining of both lung and breast MPSs (Fig. 14). Furthermore, the authors pointed out that these architectural differences (e.g., loss of defined crypt structure, tumor cells crossing the basement membrane and infiltrating the ECM) defined different grades of invasiveness in the MPSs.

**Fig. 15:**
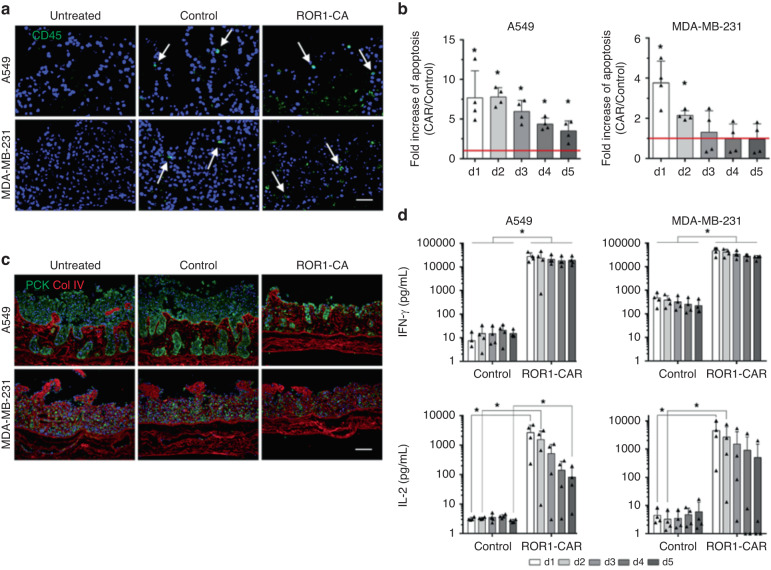
ROR1-CAR T cells migrate into tumor tissue and proliferate in static 3D culture. ROR1-CAR T cells migrate into tumor tissue and induce tumor cell lysis in dynamic 3D culture. **a** Immunofluorescence staining of CD45 (green) on paraffin sections of dynamic tumor models treated with control or ROR1-CAR T cells. White arrows mark T cells that had migrated into the tissue matrix. **b** Immunofluorescence double staining of PCK (green) and Col IV (red) on paraffin sections of untreated dynamic tumor models as well as tumor models treated with CD4+ and CD8+ control T cells or CD4+ and CD8 + ROR1-CAR T cells. **c** Quantification of the apoptosis rate in ROR1-CAR T cells that had accumulated during 5 days of treatment. Apoptosis was measured with M30 ELISA from supernatants collected at the indicated time points, and the results are presented as fold change compared with respective control T-cell treatment (red line). **d** ELISA-based quantification of IFN-γ and IL-2 levels in supernatants collected over time from dynamic tumor models treated with T cells for 5 days. Adapted from Wallstabe et al.^127^

Then, the authors tested the antitumor efficacy of ROR1-CAR T cells with their MPSs. ROR1-CAR T cells actively entered the arterial medium flow in the dynamic culture setup and adhered to and invaded the tumor mass. Thereafter, ROR1-CAR T cells were able to penetrate the tumor cell mass in the MPSs (CD45 staining in Fig. 15a) and induce apoptosis in both tumor cell types, most effectively within the first few days (Fig. 15b). The reduction in the tumor mass size and related architectural changes were visible via histology as well (Fig. 15c). The authors observed a marked change in INF-γ and IL-2 secretion levels via ELISAs (Fig. 15d); both of these factors are produced and consumed by CAR-T cells, thereby playing a critical role in the effector function of these cells. Overall, the authors concluded that ROR1-CAR T cells were effective at penetrating a tumor mass and killing tumor cells^127^.

Another study reported by Lee et al.^128^ described an MPS used to study T-cell cytotoxicity against hepatocellular carcinoma (HSCC) caused by hepatitis B virus (HBV). Due to HBV infection, HCC cells express virus-derived antigens that can be recognized by T cells and thus increase T-cell cytotoxicity. This strategy for increasing cytotoxicity offers promising therapeutic potential for treating patients with HBV-HCC. Therefore, the performance of HBV antigen-specific T cells in the suppressive environment of HCC was assessed and reported in this paper. The authors used a triple-channel microdevice to culture HBV-HCC organoids and other stromal cells (e.g., monocytes) in a 3D matrix in the central channel. HBV antigen-specific T cells were then perfused through the lateral channels, and tumor infiltration of immune cells and tumor cell killing were analyzed. Using this MPS, the authors demonstrated the increased cytotoxic capacity of HBV antigen-specific T cells compared with naive T cells but also observed that tumor-educated monocytes suppressed T-cell cytotoxic activity through the PD-1/PD-L1 pathway.

As previously mentioned, an important mechanism of inhibition of T and NK cell cytotoxicity is tumor-derived hypoxia^34^. Thus, identifying new strategies to generate T and NK cells resistant to tumor hypoxia may greatly increase their efficacy. With this goal in mind, Ando et al.^129^ developed a microdevice that included a circular chamber into which a 3D hydrogel with HER-2^+^ human ovarian cancer cells (SKOV-3) was applied. The circular chamber was surrounded by a channel that was perfused with medium to provide oxygen and nutrients to the central chamber. The microdevice allowed users to manipulate oxygen diffusion through the microdevice, allowing them to generate gradients of oxygen and nutrients or only nutrients. Thus, the authors evaluated the effect of oxygen and nutrient gradients (independently and in combination) on T cells and CAR T cells transduced with scFv 4D5 (anti-HER2 antibody). The results demonstrated that hypoxia led to a greater decrease in T and CAR T-cell cytotoxicity than nutrient gradients. Although the authors characterized the oxygen gradients in detail, more in-depth studies are required to pinpoint the specific function and molecular mechanisms driving hypoxia-induced immunosuppression. Future studies may be designed to explore whether hypoxia limits tumor killing by decreasing T-cell infiltration, proliferation, or cytotoxic capacity, all of which might explain hypoxia-induced immunosuppression.

Pavesi et al.^130^ used a triple-channel device to study the role of immunosuppressive and activator cytokines and oxygen concentration in CAR T cells. The authors cultured hepatocellular cancer (HCC) organoids in a collagen hydrogel and central channel. After collagen polymerization, they perfused CAR T cells engineered with a repertoire of CAR formulations. The authors used the model to identify the optimal CAR formulation based on tumor killing and cytokine secretion rates. Additionally, the authors supplemented the model with inflammatory (e.g., IL-2) and immunosuppressive cytokines/proteins (e.g., mTOR inhibitors) to study the effect of the environment on their CAR T-cell formulations^130^.

Similarly, Aung et al.^131^ used photopatterning to culture multiple cell types in concentric gelatin circular layers, generating an in vitro culture with a dense MCF7 breast cancer cell organoid at the center surrounded by monocytes (THP-1 cells) and endothelial cells in the outer layer. The authors used this MPS to study the effect of monocytes on T-cell recruitment to the cancer organoid. The embedded spheroids recruited more T cells compared than a similar culture where breast cancer cells were seeded to create a 3D model. Interestingly, the authors suggested that this increase in migration was due to the hypoxic environment observed in the organoid, which was the trend opposite that observed by Lee et al.^128^. This study suggested a role for monocytes in promoting T-cell recruitment, which seems contradictory to the findings reported by Ando et al.^129^. These apparently counterintuitive observations highlight the complex and sometimes dual role of monocytes in tumor immunosurveillance and call for further studies in this field.

MPSs for immuno-oncology applications commonly rely on isolating cells from blood and tissue samples (e.g., tumor biopsy) and then assembling them in the desired geometry to leverage predictable fluid behaviors at the microscale. However, this process inherently destroys the tissue microenvironment, which may modify the response of tissue-resident immune cells. Thus, other microfluidic models have focused on directly culturing tissue samples while preserving the environment and the tissue-resident immune population. Beckwith et al. used this approach to culture patient-derived tumor samples and evaluated the response to immune checkpoint inhibitors (ICIs) by fluorescence microscopy. The authors studied the response of resident lymphocytes in biopsied tumor tissue to immunotherapeutic agents in the context of a perfused tumor microenvironment. The microfluidic platform consisted of a 3D-printed, transparent and noncytotoxic substrate. This study illustrated how microfluidic models can be applied for precision medicine, providing clinically relevant information faster than other protocols that require tissue digestion and cell isolation^132^.

T-cell bispecific antibodies (TCBs) are engineered antibodies that simultaneously recognize T cells and tumor cells, thereby increasing tumor cell recognition by T cells and the formation of immune synapses. Thus, they hold interesting potential as new therapeutics to increase the antitumor immune response. However, TCBs commonly cause off-tumor tissue toxicity due to low levels of tumor antigens in healthy tissues (e.g., EGFR). Thus, the development of efficient and safe TCBs is challenging and requires thorough testing in systems that capture the antigen expression profile observed in human tissues and tumors. Kerns et al.^133^ used a dual-chamber microfluidic device to evaluate experimental TCBs designed against lung and colon cancer. They used in vivo target expression and toxicity data from TCBs targeting folate receptor-1 (FOLR1) or carcinoembryonic antigen (CEA). FOLR1 is overexpressed in many solid epithelial tumors, such as ovarian, lung, and breast cancers, but it is also expressed to a lesser extent in normal epithelial cells, such as those found in the lung and kidneys. Based on sensitivity to major factors of target expression and antibody affinity, they discovered that the lung and intestine chips mimicked and could be used to predict target-dependent TCB safety liabilities. These unique methods may contribute to furthering our understanding of how tailored therapeutic antibodies work as well as for determining safety profiles and potential adverse outcomes^133^.

Al-Samadi^134^ used a five-channel microdevice to evaluate immune checkpoint inhibitors and immunomodulatory agents to increase the immune response against patient-derived HNSCC. More specifically, this author evaluated the potential use of IDO-1 inhibitors in combination with anti-PD-L1 antibodies. IDO-1 is an enzyme involved in tryptophan metabolism, which is essential for T and NK cell effector functions. Multiple studies have shown that tumors commonly show upregulated IDO-1 expression, which inhibits T and NK cell responses. The authors cultured HNSCC cell lines and patient-derived samples in a central channel in a collagen hydrogel and used the other lateral channels to perfuse immune cells isolated from healthy donors alone or in combination with anti-PD-L1 antibodies or IDO-1 inhibitors. Their results showed that both anti-PD-L1 antibodies and IDO-1 inhibitors increased immune cell penetration and cytotoxicity in a patient-specific manner. In future studies, this platform can be used to evaluate combinations of PD-L1 antibodies and IDO-1 inhibitors.

Another MPS application to evaluate the efficacy of immunotherapies has been aimed at improving outcomes in glioblastoma multiforme (GBM), a common and aggressive brain tumor. GBM poses daunting challenges to successful immunotherapy, including the presence of the blood‒brain barrier (BBB), which can limit immune cell and drug penetration, and a highly immunosuppressive microenvironment. Thus, GBM patients are in dire need of more efficient therapies. Cui et al.^135^ isolated patient-derived GBM cells and allogenic PBMCs and cultured them in a three-channel microdevice with a circular central microchamber surrounded by two side channels. In this central chamber, the team cultured patient-derived GBM organoids in a 3D brain-mimicking hyaluronan (HA)-rich Matrigel extracellular matrix with embedded macrophages. Next, the researchers coated the lateral chambers with human brain microvascular endothelial cells to produce an MPS mimicking the BBB. Finally, they perfused allogenic CD8 T cells through the other channels to monitor T-cell extravasation, migration, and tumor effector capacities. This report demonstrated that GBM cells generated an immunosuppressive environment characterized by elevated PD-L1 and TGF-b1, IL-10, and CSF-1 secretion rates. The environment of their MPS induced the migration of macrophages and induced macrophage polarization toward a tumor-associated and immunosuppressive phenotype (CD68^+^, CD163^+^). Real-time analysis with the MPS showed that tumor-associated macrophages (TAMs) inhibited the recruitment of CD8^+^ T cells. Next, the authors used their platform to evaluate the efficacy of experimental therapies combining PD-1 inhibitors (e.g., nivolumab) with other immunomodulatory agents, such as the CSF-1R inhibitor BLZ945, which ablated CD163+ TAMs and increased CD8 + T-cell cytotoxicity against GBM cells. Additionally, the authors compared these responses across different GBM cell subtypes (i.e., classical, proneural, and mesenchymal type), demonstrating the potential use of these platforms for precision medicine in brain cancer^135^.

Finding strategies to prevent tumor cell immune escape remains a pressing need to improve the outcomes of immunotherapy. However, a hurdle to improved outcomes is that traditional assays used to quantify immune cell proliferation, death, or activation commonly rely on destructive techniques (e.g., ATP-content analysis) or time-consuming protocols that are not readily scalable^136^. Microfluidic devices are highly compatible with integrated sensors, which can streamline the data collection and analysis process. Wu et al.^137^ developed an optofluidic device that included two culture chambers coated with barcoded antibodies and surface-enhanced Raman scattering (SERS) nanoprobes. Tumor cells were cultured in one of these chambers to allow them to secrete immunosuppressive cytokines. Immune cells were placed in the adjacent chamber and treated with a library of cancer drugs and inhibitors. A SERS-assisted 3D barcode immunoassay is a nondestructive technique to monitor the effect of these drugs and inhibitors on cytokine production and their effect on immune cells in real-time. As opposed to traditional methods used in precision medicine, this platform can reduce the screening time by identifying the best drug and immunosuppression inhibitor combination for each patient^137^.

## NK cell cytotoxicity

NK cell-based immunotherapies also hold great potential and arguably confer advantages to T cells since they do not mediate graft-versus-host disease. Allogeneic therapies with nonengineered NK cell therapies have entered the clinical trial stage, but their CAR-modified versions may prove more efficacious after further development^136^. Thus, most NK cell-based immunotherapies must be considerably optimized before they can be used in the clinic, and the studies of these immunotherapies may benefit from preclinical assays using MPSs to evaluate the ability of immunotherapies to overcome tumor cell immune evasion mechanisms and, in turn, increase antitumor effector capacities.

With this goal in mind, Giannattasio et al.^138^ cultured cervical carcinoma cell lines into 3D spheroids and analyzed their sensitivity to primary IL-2-activated NK cells. This study demonstrated that NK cells penetrated tumor spheroids. Further molecular analysis revealed that when grown as spheroids, tumor cells shed antigens involved in NK cell-mediated cytotoxicity, rendering tumor spheroids more resistant to NK cell surveillance^138^. This study highlighted antigen shedding as an additional mechanism underlying the immune escape of solid tumor cells; this information may be used to increase the effectiveness of NK cell-based therapy^139^.

As mentioned, the TME can limit NK cell performance in a manner that resembles T-cell exhaustion, although it is unclear whether NK cell exhaustion mechanisms are similar to those in T cells. To improve our understanding of NK cell exhaustion mechanisms, Christakou et al.^140^ developed a microfluidic device containing a microwell array where Hep2G cells were seeded and aggregated into a single spheroid in each well using static ultrasound waves. The main advantage of their platform was the capacity to control the size and shape of the tumor spheroid generated by modifying the ultrasonic wave. Once a spheroid was formed, the authors added NK-92 cells at several densities to measure NK-92 cell penetration of spheroids and tumor cell killing effects^140^. Although the authors did not explore the microenvironmental factors that may drive NK cell exhaustion in vivo, the platform represented an early proof-of-concept for the study of NK cell antitumor effector capacities.

T and NK cell effector capacities against tumor cells are highly heterogeneous, with many factors contributing to this heterogeneity, such as the T-cell repertoire or killer cell immunoglobulin-like receptor (KIR) expression. However, most of the techniques commonly used to measure cytotoxicity are used with T or NK cells in bulk. Alternative approaches that can produce single-cell results of effector capacity would be beneficial to optimize T and NK cell performance against tumors. Thus, microfluidic devices with microwells or microdroplets can be used to combine individual immune and tumor cells. Sarkar et al.^141^ used this approach to identify NK cell clones with the highest cytotoxic effect against tumor cells (multiple myeloma). Using their microfluidic platform, the authors demonstrated that even cell populations traditionally considered relatively homogeneous, such as NK-92 cells, exhibit large heterogeneity in terms of cytotoxic capacity. Although the authors did not explore the molecular mechanisms contributing to higher/lower cytotoxicity in their study, their results provide a valuable example of how these technologies an contribute to the generation of more efficient immune cell formulations^141^.

Antibody-dependent cell cytotoxicity (ADCC) is an adaptive immune response largely mediated by NK cells based on using specific antibodies that can recognize and mark target cells for killing. These cells are recognized based on their expression of specific tumor- or pathogen-derived antigens on their surface. ADCC can be used to aid cancer cell recognition and the effector capacities of NK cells. In fact, several clinical trials are exploring the use of adoptive cell therapy in combination with antibodies such as anti-PD-1 (pembrolizumab), CTLA-4 (ipilimumab), HER-2 (trastuzumab), or EGFR (cetuximab). ADCC has also been explored as a strategy to boost NK cell effector capacities in several MPS studies. Nguyen et al. used a five-channel device to demonstrate the potential of trastuzumab (i.e., anti-HER-2 antibody) to trigger ADCC in HER-2^+^ breast cancer. One of the most relevant findings of this study was the antagonist role of CAFs in trastuzumab-induced tumor-immune interactions and ADCC^142^. Their results demonstrated that CAFs hindered trastuzumab-mediated ADCC, partially protecting tumor cells.

To explore the synergy between NK cell-based immunotherapy and modified antibodies such as immunocytokine (e.g., IL-2 coupled antibody), Ayuso et al.^143^ developed an MPS that with cultured breast cancer spheroids in a 3D matrix flanked by lateral biomimetic blood vessels to explore the synergy between NK cell-based immunotherapy and modified antibodies such as immunocytokines (e.g., IL-2 coupled antibody). The MPS revealed the generation of a hypoxic region inside of the tumor spheroid, and more importantly, cell‒cell junctions created a barrier effect that severely limited the penetration of immunocytokine. Thus, immunocytokine remained at the tumor periphery after multiple days in culture despite the migratory capacity exhibited by NK cells, which reached the core of the tumor after a few hours. Thus, ADCC was severely limited and caused only a moderate increase in NK cell killing capacity at the outer layers of the tumor spheroid. Furthermore, confocal microscopy revealed that tumor cells showed the capacity to endocytose the membrane-bound antibody via intracellular lipid vesicles, arguably protecting the cells from NK cell ADCC in the long term. Researchers can leverage these and other strategies to reduce tumor antigen shedding and increase antibody stability on the tumor cell membrane^144^.

**Fig. 16:**
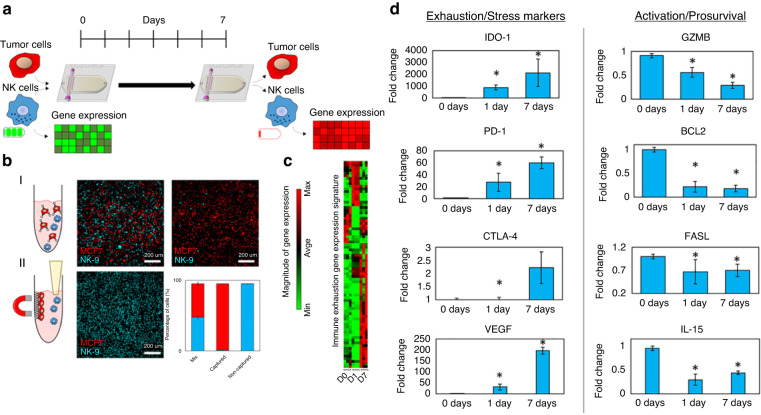
MPS that recapitulates depleted environments and NK cell exhaustion. **a** Schematic representation of an experiment measuring immune cell exhaustion. **b** Schematic showing NK cell separation. Tumor cells attach to magnetic beads, thus isolating NK cells in suspension. Confocal images represent captured MCF7 cells (in red) and isolated NK cells (in blue). **c** Cluster graph depicting gene expression after 0, 1, and 7 days. **d** Bar graphs show the up-/downregulation of exhaustion markers and activation/pro-survival genes. Adapted from Ayuso et al.^146^

An extensive body of literature has revealed the relevance of immune exhaustion during tumor evolution^145^. Immune cells within the TME commonly exhibit progressive loss of function, characterized by decreased proliferation and cytotoxicity, which in turn lead to tumor tolerance. Multiple MPSs have been developed to study the molecular mechanisms driving immune cell exhaustion within solid tumors and to evaluate new therapeutics that prevent/revert immune cell exhaustion. In particular, we developed an MPS with cultured breast or colon cancer cells (i.e., MCF-7, HCT-116) embedded in 3D collagen hydrogel^146^ (Fig. 15a). As described in other studies^80,83^, the hydrogel included a tubular compartment lined with endothelial cells to mimic the structure of blood vessels. Culture medium perfused through the vessel alone established nutrient and pH gradients across the hydrogel. Therefore, the tumor cells located far from the biomimetic blood vessel died, forming a necrotic core, whereas cells next to the vessel remained viable. The authors perfused NK cells (NK-92) through the biomimetic vessel, demonstrating the capacity of NK cells to infiltrate the tumor. Further isolation of NK-92 cells allowed genetic profiling to be performed, and the results were used to evaluate the effects of microenvironmental conditioning on these cells (Fig. 15b). More importantly, the results demonstrated that NK cells gradually lost their cytotoxic capacity as they penetrated deep into the tumor Fig. 16. Molecular analysis revealed that NK cells became exhausted as they infiltrated across the tumor model, showing higher levels of PD-1, CTLA-4, or IDO-1 and downregulating prosurvival and activation genes such as GZMB or BCL-2 (Fig. 15c, d).

**Fig. 17:**
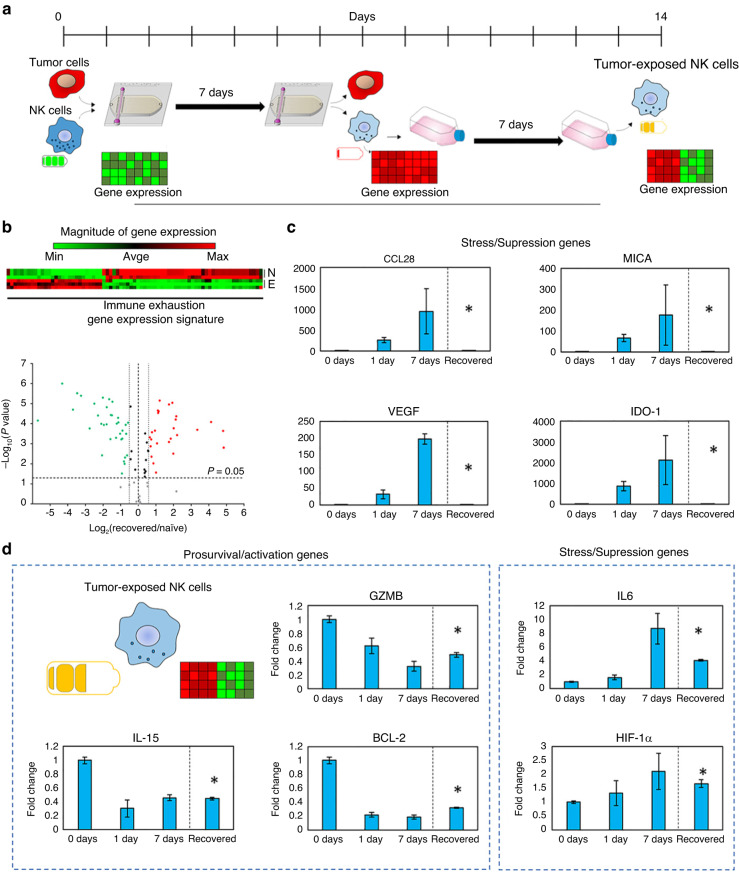
NK cells partially recover from MPS-induced exhaustion. **a** Schematic representation of an experiment performed to measure immune cell exhaustion. **b** Clustergram showing changes in gene expression in the tumor-on-a-chip (ToC) compared with those in the petri dish cultures. **c**, **d** Bar graphs depicting the change in gene expression of exhaustion markers and activation/prosurvival genes in the tumor-on-a-chip device condition versus the petri dish condition. Reproduced from Ayuso et al.^146^

NK cells retrieved from the model described in Ayuso et al.^146^. were subcultured to evaluate their capacity to recover from tumor-induced immune cell exhaustion (Fig. 17a). However, NK cells did not revert to their original phenotype after they were removed them from the MPS; in contrast, they showed molecular signs of immune cell exhaustion and reduced proliferation, migration, and cytotoxicity (Fig. 17b–d). This experiment demonstrated that nutrient and pH gradients play a critical role in NK cell exhaustion but did not lead to the identification of specific metabolites driving NK cell exhaustion, highlighting the need for more in-depth studies that may lead to the identification of targetable pathways.

## Microfluidic devices to improve immune cell genetic engineering

T cells exhibit highly heterogeneous TCR expression, leading to the generation of a large TCR repertoire with varying degrees of specificity against tumor antigens. Thus, adoptive cell therapies are commonly based on T-cell clones with the highest reactivity against tumor-specific antigens. T-cell clone selection is a time-consuming process that involves the culture of large amounts of T cells in single-cell platforms to identify the most efficient clones. Thus, researchers have leveraged microphysiological platforms to streamline the process, improving the efficacy of the monitoring process and clone selection^147^. These platforms commonly rely on droplet microfluidics, cell traps, and well-plate designs to coculture one T-cell clone with one tumor cell. Microscopy and fluorescent reporters are used to detect T-cell engagement and cytotoxicity, and the most reactive clones are isolated using laser sorting or cavitation.

CAR T-cell generation techniques require aggressive manipulation of immune cells to deliver a CAR-expressing vector into a T cell, which significantly limits process efficiency and delays product development. Sharei et al.^148^ demonstrated that human cells, including immune cells, develop transient microscopic pores in the cell membrane after flowing through a narrow microfluidic channel. Thus, they used this behavior to deliver expression vectors (e.g., CARs) into human T cells. Their results demonstrated that this transduction method was very efficient and induced minimal toxicity and few off-target effects compared with other methods commonly used for CAR T-cell generation (e.g., electroporation).

CAR T cell applications against cancer cells are promising potential therapeutics. However, therapeutic T-cell efficiency is limited by the endogenous T-cell response. Several aspects of natural response programs may be harmful, while others, such as a program to overcome tumor immunosuppression, are lacking. Therefore, if precisely designed immune cell responses can be specially triggered, then the effectiveness and safety of therapeutic cells can be enhanced. Synthetic Notch receptors induced transcriptional activation in response to recognition of user-specified antigens. They have been used in primary T cells because they can be used to induce personalized cytokine secretion profiles, biased T-cell development, and local delivery of nonnative therapeutics (i.e., antibodies in response to antigens). This technology based on microfluidic platforms can be used to recognize and remodel local microenvironments associated with a variety of disorders^149^. MPSs may be alternative tools that complement traditional in vitro and in vivo strategies to accelerate the development and evaluation of new immunotherapies, with a few MPSs applied for high-throughput screening^150–152^.

## Concluding remarks: remaining challenges and potential opportunities

Immunotherapy has improved outcomes for patients with a variety of malignancies. However, there are still some critical challenges that hinder the efficacy of immunotherapies, such as antigen loss, poor immune recruitment, or immune cell exhaustion. In recent years, researchers have explored the potential use of microphysiological systems to overcome these challenges. As discussed, microphysiological systems excel at mimicking tissue architecture, and are thus versatile tools to capture critical features of tumor cell–immune cell interactions. Additionally, microphysiological systems can offer increased throughput compared with animal models or even traditional well plates. In the early 2010s, these platforms were focused primarily on proof-of-concept experiments, but in recent years, namely, the 2010s and early 2020s, there has been a rise in the number of studies focusing on leveraging these systems to answer biologically relevant questions, providing more in-depth molecular and cellular results. Nevertheless, despite these provocative studies, microphysiological systems for immunotherapy are still limited, and some obstacles are difficult to address. There is still an ongoing debate about the complexity needed before in vitro models can accurately predict patient response. Traditionally, genetic factors have been at the forefront of immunotherapy predictors (e.g., PD-1 expression). However, recent studies have highlighted the plethora of additional factors that affect immunotherapy outcome, including metabolic parameters, the stromal compartment, the vasculature, the nervous system, and even the microbiome. Despite the superior control of microphysiological systems, including all these parameters in platforms in vitro may rapidly the system complexity, making it extremely challenging to manipulate. Thus, arguably, additional studies are still needed to decipher what is the degree of complexity needed for each type of malignancy. We believe that, now that microphysiological systems seem to be moving beyond proof-of-concept experiments, the technology is mature enough to explore its full potential in the field of immunotherapy in the future.
